# A Validated Smartphone-Based Assessment of Gait and Gait Variability in Parkinson’s Disease

**DOI:** 10.1371/journal.pone.0141694

**Published:** 2015-10-30

**Authors:** Robert J. Ellis, Yee Sien Ng, Shenggao Zhu, Dawn M. Tan, Boyd Anderson, Gottfried Schlaug, Ye Wang

**Affiliations:** 1 School of Computing, National University of Singapore, Computing 1, 13 Computing Drive, Singapore, 117417, Singapore; 2 NUS Graduate School for Integrative Sciences and Engineering, 28 Medical Drive, Singapore, 117456, Singapore; 3 Department of Rehabilitation Medicine, Singapore General Hospital, Outram Rd, Singapore, 169608, Singapore; 4 Department of Neurology, Beth Israel Deaconess Medical Center and Harvard Medical School, 330 Brookline Avenue, Palmer 127, Boston, MA, 02215, United States of America; University of Western Ontario, CANADA

## Abstract

**Background:**

A well-established connection exists between increased gait variability and greater fall likelihood in Parkinson’s disease (PD); however, a portable, validated means of quantifying gait variability (and testing the efficacy of any intervention) remains lacking. Furthermore, although rhythmic auditory cueing continues to receive attention as a promising gait therapy for PD, its widespread delivery remains bottlenecked. The present paper describes a smartphone-based mobile application (“SmartMOVE”) to address both needs.

**Methods:**

The accuracy of smartphone-based gait analysis (utilizing the smartphone’s built-in tri-axial accelerometer and gyroscope to calculate successive step times and step lengths) was validated against two heel contact–based measurement devices: heel-mounted footswitch sensors (to capture step times) and an instrumented pressure sensor mat (to capture step lengths). 12 PD patients and 12 age-matched healthy controls walked along a 26-m path during self-paced and metronome-cued conditions, with all three devices recording simultaneously.

**Results:**

Four outcome measures of gait and gait variability were calculated. Mixed-factorial analysis of variance revealed several instances in which between-group differences (e.g., increased gait variability in PD patients relative to healthy controls) yielded medium-to-large effect sizes (eta-squared values), and cueing-mediated changes (e.g., decreased gait variability when PD patients walked with auditory cues) yielded small-to-medium effect sizes—while at the same time, device-related measurement error yielded small-to-negligible effect sizes.

**Conclusion:**

These findings highlight specific opportunities for smartphone-based gait analysis to serve as an alternative to conventional gait analysis methods (e.g., footswitch systems or sensor-embedded walkways), particularly when those methods are cost-prohibitive, cumbersome, or inconvenient.

## Introduction

The expected number of individuals living with Parkinson’s disease (PD) will rise sharply by the year 2030, doubling the number of patients living with the disease in the year 2005 to more than 9 million [1]. With much of this increase to be found in rapidly growing countries with still-developing economies such as Brazil, China, and India [2], existing methods for managing the various challenges of PD faced by both individual patients (mental, physical, social, financial) and the medical community (diagnostic methods, therapy delivery) may prove difficult to scale up.

One of the most serious challenges in dealing with the progression of PD is an increase in gait disturbances. “Episodic” disturbances include periods of freezing, festination, or initiation hesitation [3,4]. “Continuous” disturbances affect the step-to-step spatiotemporal dynamics of gait, resulting in increased spatiotemporal gait variability (GV) (for extensive discussions, see [5–7]). The most prevalent outcome measures of GV (for reviews, see [8,9]) are second-moment statistics (i.e., standard deviation or coefficient of variation) of a series of step or stride durations or lengths. Second-moment statistics require precise information about *individual* gait events (rather than averaged gait events). As such, they are both statistically and conceptually dissociable from first-moment (i.e., mean-based) statistics [6], as supported by large-*N* factor analytic studies [10–12].

Several classic findings regarding PD and GV have been reported (for detailed reviews, see [5,6]). PD patients show increased GV relative to age-matched healthy elderly (HE) individuals [13–16], particularly when in a dopamine-deplete (off-medication) state [17,18] or when they perform a concurrent cognitive or motor task [19–21]. Conversely, GV can be reduced in PD through the use of external sensory stimulation; in particular, *rhythmic auditory cueing* (RAC) paradigms (for reviews, see [22–26]). The motor system—from locomotion to manual coordination to speech articulation—is highly adept at synchronizing or entraining to auditory rhythms (e.g., a ticking metronome, or music with a steady beat); an affordance of the intimate auditory–motor pathways in the human brain (for reviews, see [27–29]). When PD patients attempt to synchronize their heel strikes with the auditory beat, however, they show improvements in both first-moment [22,23,26] and second-moment [15,16,21,30] outcome measures of gait.

The significance of GV, however, extends beyond differences between PD and HE or reductions during an RAC paradigm. Importantly, individuals with higher-than-normal GV—both in PD and more broadly—are at increased risk of falling [6,16]; this association has been found using both retrospective [31] and prospective [12,32,33] designs. The consequences of a fall (including a high rate of serious injury [34]) extend beyond the event itself, feeding into a cycle involving fear of falling, immobilization, social isolation, depression, cognitive decline, and increased mortality [3].

In theory, if higher-than-normal GV were detected (e.g., if a GV assessment were incorporated into a regular physical examination), preventive steps (from mental strategies to gait training [35,36]) could be taken to help mitigate fall risk [37,38]. An ideal assessment system would contain three core components: the sensing hardware which records the subject’s movement, the analysis software which translates the recorded signal into an outcome measure, and a display unit which communicates the value of that outcome measure. Numerous *component-based systems*, assembled from third-party sensor, processor, and/or display units, are available. Second-moment outcome measures of GV are most frequently obtained from detected heel contacts (as reviewed in [8,9]), either using either using pressure sensors (footswitches) affixed to the heel [13, 15–17, 20, 30–32, 39, 40] or pressure sensors embedded in a rollable walkway [10–12,14,18,21,33,41,42]. Other measurement approaches (reviewed in [43–45]) center around the use of an inertial measurement unit (comprising a tri-axial accelerometer and/or tri-axial gyroscope) affixed to the torso [46–49], feet [50–54], or multiple locations [55–57], and which compute outcome measures from a series of heel strike “analogues” (i.e., accelerometer waveform events associated with actual heel strikes [58,59]).

With the advent of ubiquitous and powerful smartphones (which contain an inertial measurement unit, a processing core, and a touchscreen), proposed *self-contained systems* for gait analysis have become more frequent [60–67] (see Table 1), including our own recent investigation [68]. Smartphone-based assessments in PD—including, but not limited to gait—offer numerous potential benefits: in terms of cost savings, portability, customizability, patient tolerance, and deployment scalability [2,69,70]. In reviewing this literature, however, three limitations become apparent.

**Table 1:** Summary of key features of prior studies of smartphone-mediated gait analysis.

			Recording parameters		Outcome measures derived from		Concurrent validity obtained for	
First Author (Year)	Group (N)	Age: M (SD)	Devicelocation	SF	Step/stride times	Step/stride lengths	Step/stride times	Step/stride lengths
Chan (2011) [60]	HY (1^a^)	n/a	Left pocket	100	Stride Δ_M_	—	—	—
How (2013) [61]	HY (1^a^)	n/a	Front waist	60	—	—	Footswitches	—
LeMoyne (2011) [62]	HY (1^a^)	n/a	Left ankle	100	Stride Δ_M_	—	Footswitches	—
Mellone (2012) [63]	HE (49)	59 (16)	Lower back	50	Step Δ_M_, Δ_SD_	—	Accel.	—
Nishiguchi (2012) [64]	HY (30)	20.9 (2.1)	Lower back	33	—	—	Accel.	—
Palmerini (2011) [71]	HE (49)	58.9 (16.5)	Lower back	50	Step Δ_M_, Δ_CV_	—	—	—
Yamada (2011) [66]	RA (39) / HE (20)	65.9 (10) / 69.1 (5.8)	Lower back	33	Step Δ_M_, Δ_CV_	—	Accel.	—
Yang (2012) [67]	HY (13)	23–36	Lower back	100	Cadence	Step Δ_M_ ^b^	Accel.	—
Zhu (2014) [68]	PD (10)	66.3 (7.8)	Front waist	100	Step Δ_M_, Δ_CV_	Step Δ_M_, Δ_CV_	Footswitches	GAITRite
[Present study]	PD (12) / HE (12)	65.0 (8.4) / 63.1 (7.8)	Front waist	100	Step Δ_M_, Δ_CV_	Step Δ_M_, Δ_CV_	Footswitches	GAITRite

*Abbreviations*: Accel.: conventional accelerometer; Δ_M_: mean inter-event interval; Δ_SD_: standard deviation of inter-event intervals; Δ_CV_: coefficient of variation of inter-event intervals; HE: healthy elderly; HY: healthy young; RA: rheumatoid arthritis; PD: Parkinson’s disease; SF: sampling frequency.

^*a*^ pilot data used to illustrate algorithms or processing steps in a proof-of-concept format;

^*b*^ obtained by dividing the pre-specified walking distance by the number of detected steps.

A first limitation concerns the target sample. Most previous investigations focused either on healthy young [60–62,64,67] or healthy elderly [63,65] individuals. Step detection algorithms trained using healthy subject data may not yield accurate results when tested with PD data, due to important differences in the spatiotemporal dynamics of gait between these two populations. Conversely, the algorithms developed in our previous paper [68] were exclusively trained on PD data, and have not yet been evaluated on healthy elderly data.

A second limitation concerns the use of concurrent validation; that is, an analysis of the accuracy of outcome measures derived from the smartphone-based system relative to outcome measures derived from a conventional gait measurement system. Specifically, a key question for any novel gait analysis system is whether it accurately detects heel strike analogues in the accelerometer waveform relative to ground truth (i.e., actual heel strike events). In several previous investigations of concurrent validity, however, “ground truth” was a *second* (non-smartphone) accelerometer [63,64,66,67] rather than actual heel contacts (as in [62,68]), preventing this important question from being answered.

A third limitation concerns the examined outcome measures. Several previous investigations of smartphone-based gait analysis have examined second-moment statistics of GV [63,65,66,68], as reviewed above. With the exception of our previous report [68], however, these studies have exclusively focused on step or stride *time* measures of GV rather than step or stride *length* measures of GV. Further evaluations of this latter class of outcome measure are warranted, as they are frequently reported in the literature [10,15,18,31,42].

Thus, missing from the literature is an analysis of the accuracy of smartphone accelerometery–derived outcome measures relative to heel contact–derived outcome measures of gait and gait variability in both PD and HE.

In the present study, relative accuracy was assessed using mixed-factorial analysis of variance (ANOVA). ANOVA enables the primary “novel” source of variance (i.e., device-related measurement error) to be entered into a statistical model alongside “expected” sources of variance (i.e., differences between PD and HE and differences between self-paced versus metronome-cued conditions). By computing and comparing eta-squared (η^2^) effect sizes for each of these ANOVA model components, the relative magnitude of device-related measurement error in the context of widely-used experimental contrasts can be assessed more objectively.

## Methods

The research study described here was formally approved by the Institutional Review Board (IRB) of Bright Vision Hospital / Singapore General Hospital (approval number 2013/150/F), and conducted according to the principles expressed in the Declaration of Helsinki. Upon arrival at the testing location, all subjects were informed about the purpose of the study and provided written consent.

### 1. Participants

All subjects were recruited through the Singapore General Hospital clinics. A telephone questionnaire was first administered to screen out potential subjects who (1) are not within the age range of 40 to 85; (2) have any problems with their hearing; (3) are not able to walk independently without an aid; (4) have joint problems or other neurological, musculoskeletal or medical problems that can affect walking; (5) have sustained a fall within the past year that continues to affect their walking pattern; (6) have had surgery to implant a device (e.g., deep brain stimulation or pacemaker). Subjects who satisfied all six criteria were invited to participate in the study.

Upon arrival at the testing location, four clinical assessments were administered to PD subjects, beginning 30 to 90 minutes after standard medication intake: the complete Unified Parkinson’s Disease Rating Scale (UPDRS [72]), the modified Hoehn & Yahr stage assessment [73], the Mini Mental State Exam [74], and the Freezing of Gait Questionnaire [75]. Of these assessments, only the MMSE was administered to HE subjects. All subjects had a MMSE ≥ 24, indicating an absence of cognitive impairment.

Our initial target sample size for this study was 15 PD patients and 15 HE subjects. However, several issues during data collection resulted in incomplete data sets from one or more RAC conditions for 3 PD patients and 3 HE subjects, due to: (1) an insufficiently tight connection between SmartMOVE and the chest (i.e., a tighter elastic band was required than was available at the time); (2) data lost from SmartMOVE during file transfer; (3) poor quality contact between the footswitch sensor and the heel, yielding irregular heel strike timing data; or (4) insufficient data (particularly in GAITRite, which yields the fewest events per trial) due to patient fatigue. Because a complete set of data across all experimental conditions is required for ANOVA (i.e., no missing values are permitted), two options were available: (1) imputation of missing values or (2) casewise deletion of any subject with a missing condition. Because any form of data imputation would introduce its own new set of assumptions (e.g., as reviewed in [76]), we chose the second option for the sake of parsimony and clarity.

Thus, a final sample of 12 PD patients (5 female) and 12 HE subjects (4 female) were analyzed in this study. **Table 2** presents key demographics and scores on standard clinical ratings scales for each subject. A two-sample *t*-test revealed that the PD and HE samples did not differ in age (*p* = .577) or MMSE score (*p* = .496).

**Table 2:** Demographics of the PD and HE samples, and clinical characteristics PD sample.

	HE sample			PD sample						
	Age (yrs)	Gender	MMSSE (0 to 30)	Age (yrs)	Gender	MMSE (0 to 30)	Disease duration (yrs)	UPDRS III (0 to 56)	H & Y (1 to 5)	FOGQ (0 to 24)
	63.4	M	30	58.8	M	30	7	20	4	22
	51.0	M	30	72.1	M	30	3	33	2.5	10
	68.5	M	29	77.5	M	28	8	16	3	6
	66.1	F	29	63.4	F	24	14	24	2.5	5
	67.6	M	30	65.5	F	29	6	28	2.5	2
	64.9	M	30	81.0	M	30	2	37	2.5	1
	59.1	M	29	61.8	F	30	2	36	2	0
	60.5	F	26	63.5	M	28	4	14	1.5	2
	65.8	F	29	63.2	M	29	8	8	3	8
	79.9	M	29	49.8	M	30	5	38	2.5	9
	52.0	M	30	60.6	F	30	4	21	2.5	1
	58.2	F	30	62.3	F	28	3	22	3	10
[Mean]	63.08	—	29.25	64.96	—	28.83	5.50	24.75	2.63	6.33
[SD]	7.79	—	1.14	8.41	—	1.75	3.42	9.76	0.61	6.17

“UPDRS III” is the Unified Parkinson’s Disease Rating Scale [72] section III (lower score indicates greater motor impairment); “H & Y” is the modified Hoehn & Yahr stage assessment [73] (higher score indicates more advanced PD stage); “MMSE” is the Mini Mental State Exam [74] (lower score indicates greater cognitive impairment); and “FOGQ” is the Freezing of Gait Questionnaire [75] (higher score indicates increased freezing severity).

### 2. Walking evaluation

**Fig 1:**
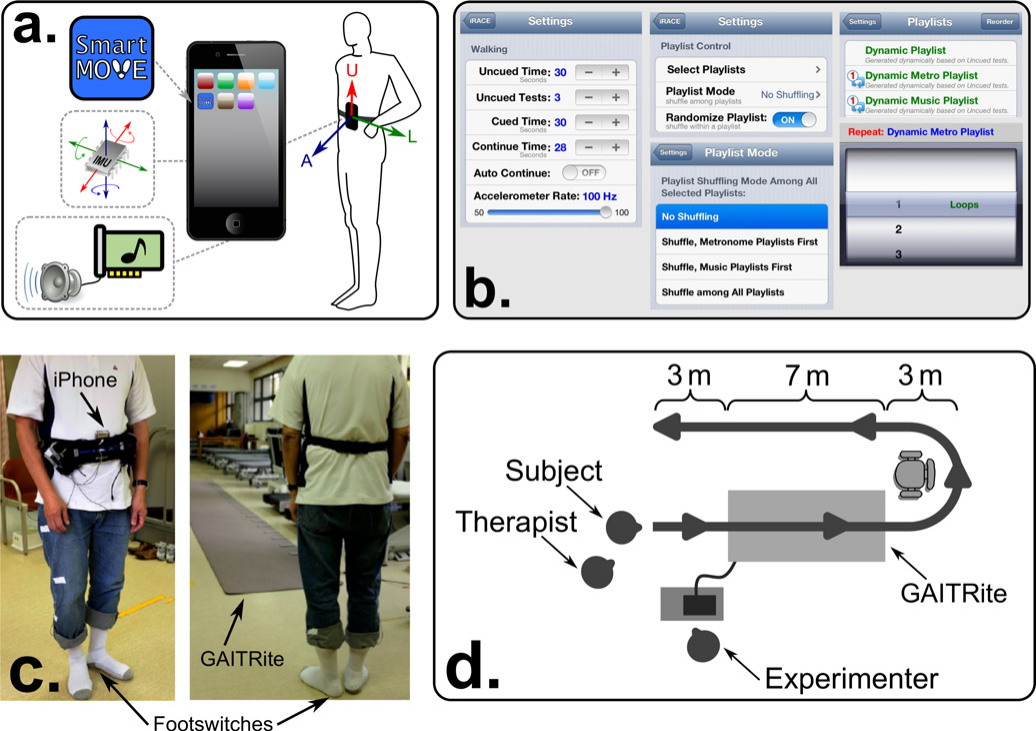
Key experimental features. The SmartMOVE mobile app (a.) utilizes the smartphone’s inertial measurement unit to record gait movements during walking. Flexible parameter settings (b.) enable precise control over testing parameters. SmartMOVE outcome measures were validated against heel-mounted footswitches and a GAITRite sensor walkway (c.) while subjects walked along a prescribed path (d.).

Fig 1 illustrates key features of the walking evaluation. The SmartMOVE app runs on an Apple iPod Touch (running iOS 6.1) and was secured at the subject’s navel using an elastic strap (Fig 1A), and positioned with the screen facing towards the subject and the audio jack facing up (so that the device’s loudspeaker was unobstructed). SmartMOVE records 6 channels of IMU data, all at 100 Hz, in the iOS device *xyz* coordinate system: tri-axial acceleration (*a*_*x*_, *a*_*y*_ and *a*_*z*_) and tri-axial gyroscopic rotation rate (ω_*x*_, ω_*y*_ and ω_*z*_). For simplicity, directions are defined with respect to the subject: anterior–posterior (AP) acceleration as *a*AP = −*az* (positive values for anterior acceleration), up–down (UD) acceleration as *a*UD = −*ay* (positive values for upward acceleration), and left–right (LR) acceleration as *a*_LR_ = *a*_*x*_ (positive values for leftward acceleration). Similarly, we define the rotation rate around the AP axis (i.e., roll) as ω_AP_ = −ω_*z*_, around the UD axis (i.e., yaw) as ωUD = −ωy, and around the LR axis (i.e., pitch) as ω_LR_ = ω_*x*_. (These labels were incorrectly stated in Section 5.1 of Zhu et al. [68]; an erratum [77] and corrected manuscript [78] are available.)

A footswitch sensor was affixed to each heel pad before fitting subjects with a layer of rubber-toed socks (to protect the sensor and provide traction). A single trial consisted of a 26-meter path, including 7-m on the GAITRite, and a single turn at the half-way point (Fig 1C). Trials were repeated until a minimum of 40 steps were recorded on the GAITRite itself, in three sequential conditions: (1) “Self-paced” (no external cue), (2) “100% RAC” (metronome tempo set at the average self-paced cadence, as determined by GAITRite), and (3) “110% RAC” (metronome tempo set 10% faster than 100% RAC). 100% and 110% relative tempos are common in the RAC literature [15,16,21,22,40,79–81], and are easily configured using SmartMOVE’s menu settings (Fig 1B). Any trial in which a subject experienced gait freezing was discarded and performed again.

### 3. Gait analysis

A nine-part preprocessing pipeline was used to translate raw SmartMOVE accelerometry data into a series of inter-step times and inter-step lengths, and is detailed in S1 Text. For a given experimental condition, “∆-series” of inter-step events (times and lengths) was derived by concatenating inter-step events across individual trials in that condition as in Lord et al. [17,82]). Two outcome measures were obtained for each ∆-series: the mean (∆_M_) and the coefficient of variation (∆_CV_, defined as 100 × ∆_SD_ / ∆_M_, where ∆_SD_ is the standard deviation of ∆-values). As noted in the Introduction, these statistics are widely used in the PD literature, including investigations of RAC [15,16,21–23,26,30], and have high test–retest reliability [83].

### 4. Quantifying device-related measurement error: ANOVA and η^2^

In seeking to validate a novel measurement device against a standard measurement device, an analysis of the magnitude of measurement error is critical. Just as critical, however, is understanding the context in which that inter-device error emerges; that is, the magnitude of measurement error *relative to* the magnitude of the target experimental effects; for example, inter-group (PD vs. HE) and inter-task (self-paced vs. metronome-cued) effects. ANOVA provides a convenient way of capturing all these sources of variance simultaneously (see S2 Text for further justification).

For each outcome measure (step time and step length Δ_M_; step time and step length Δ_CV_), three ANOVAs were performed using Statistica. Each of these analyses represents a potentially “self-sufficient” experimental question, and thus a distinct ANOVA design:

1. A *Group* (2 levels: PD and HE) × *RAC* (3 levels: self-paced, 100%, and 110%) × *Device* (2 levels: either SmartMOVE Biometrics footswitch sensors for step time outcome measures, or SmartMOVE and GAITRite sensor walkway for step length outcome measures).
2. A *Group* × *Device* ANOVA during self-paced walking to determine whether differences between PD and HE were significantly different when measured by SmartMOVE versus the novel versus the standard device.
3. An *RAC* × *Device* ANOVA for PD patient group in isolation.
4. An *RAC* × *Device* ANOVA for HE subject group in isolation.

For each ANOVA component (main effect or interaction), eta-squared (η^2^) effect sizes were computed [84, 85]. η^2^ quantifies the proportion of total variance (from 0 to 1) that is captured by a particular ANOVA component. By convention, an η^2^ ≈ .02 is considered a “small” effect, an η^2^ ≈ .13 a “medium” effect, and an η^2^ ≈ .26 a “large” effect [86, 87]. Thus, the primary purpose of these analyses is to identify those instances (if any) in which a significant main effect for *Device* or interaction with *Device* emerged, and to quantify the magnitude of *Device*-related effects (i.e., η^2^ values) relative to *Group*- or *RAC*-related effects. Instances in which “expected” sources of variance (i.e., main effects for *Group* and/or *RAC*) had substantially larger effect sizes than main effects or interactions with *Device* would indicate situations in which SmartMOVE could serve as a viable alternative to conventional gait analysis systems.

## Results

Two important “precursor” results which extend our previous report [68] should first be noted. First, accelerometer waveform peaks were confirmed to be the most temporally stable waveform analogue of actual heel strikes both PD and HE datasets, as detailed in Step 4 of S1 Text. Second, the machine learning algorithms for waveform peak identification, step length calculation, and left-versus-right foot identification were confirmed to perform with very high accuracy in the full dataset comprising PD and HE subjects, as detailed Step 5 of S1 Text.

**Fig 2:**
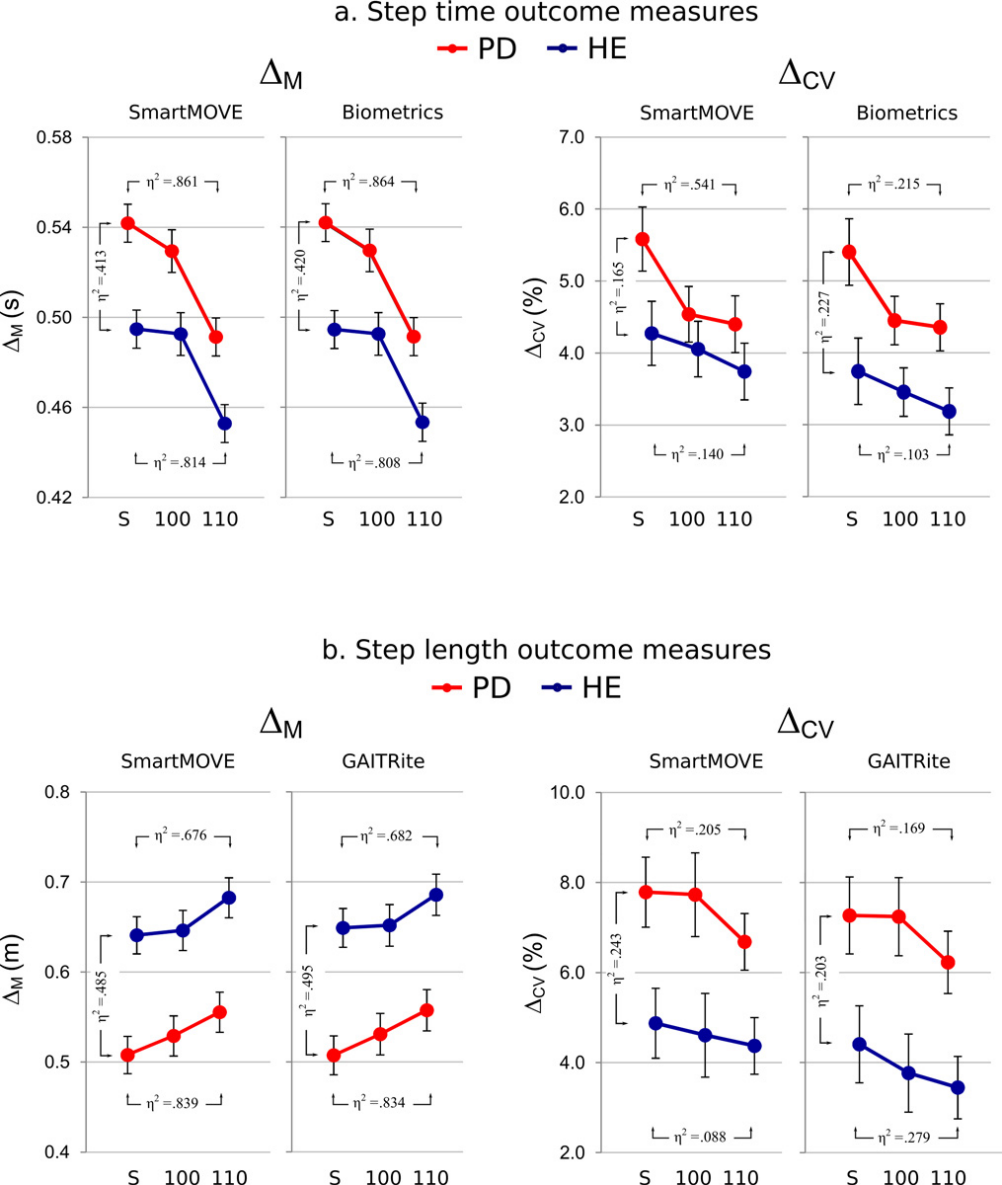
Outcome measure results. Results of the *Group* (separate lines: PD vs. HE) × *Condition* (*x*-axis: self-paced, 100% RAC, 110% RAC) × *Device* (separate panels: SmartMOVE vs. heel contact–based) ANOVAs for step time (a.) and step length (b.) outcome measures.

Next, the key results of the present study—the accuracy of SmartMOVE-derived outcome measures relative to heel contact–derived outcome measures—are presented. Fig 2 plots group-level means and standard errors for each outcome measure, as a function of group (PD in red; HE in blue), RAC condition levels (*x*-axis), and device (separate panels). η^2^ values for three simple effects (PD versus HE during self-paced walking; self-paced versus 110% RAC in PD; self-paced versus 110% RAC in HE) are highlighted for each outcome measure on Fig 2. S1 Table and S2 Table provide the group means and error bars for step time and step length data, and S1 Dataset and S2 Dataset contain the individual subject outcome measure data used to perform the step time and step length ANOVAs. The four ANOVAs described in Section 4 of the Methods are presented in succession.

### 1. *Group* × *RAC* × *Device* ANOVA

The three-factor ANOVA enables a comparison of inter-group, inter-condition, and inter-device variance for each outcome measure. Table 3 and Table 4 present the step time and step length ANOVA statistics, respectively. As expected, significant group differences emerged in all four outcome measures: relative to HE subjects, PD patients walked with slower steps (step time Δ_M_), shorter steps (step length Δ_M_), and increased step time and step length variability (Δ_CV_). Additionally, relative to self-paced walking, metronome-cued walking set to 110% of self-paced cadence yielded significantly faster walking with longer steps and less step-to-step variability. Effect sizes (η^2^ values) for *Group* were typically much larger than for *RAC*, indicating a greater separation of outcome measure values.

**Table 3:** Statistics associated with the *Group* × *RAC* × *Device* ANOVA for step time outcome measures.

	Δ_M_				Δ_CV_			
Effect	*SS*	*F*	*p*	η^2^	*SS*	*F*	*p*	η^2^
Device	3.57 × 10^−7^	2.82	.108	< .001	4.03 × 10^0^	5.01	**.036**	.013
Group	5.99 × 10^−2^	12.41	**< .001**	.248	3.93 × 10^1^	5.51	**.028**	.125
Group × Device	5.96 × 10^−8^	.47	.500	< .001	2.34 × 10^0^	2.34	.140	.006
RAC	5.89 × 10^−2^	79.95	**< .001**	.243	1.79 × 10^1^	6.04	**.005**	.057
RAC × Device	7.71 × 10^−7^	2.18	.125	< .001	2.02 × 10^−2^	.06	.939	< .001
Group × RAC	7.59 × 10^−4^	1.03	.366	.003	3.65 × 10^0^	1.23	.302	.012
Group × RAC × Device	1.13 × 10^−6^	3.19	.051	< .001	5.31 × 10^2^	.17	.848	< .001

“SS” is the portioned sums of squares for each ANOVA term. Significant *p*-values are highlighted in bold.

**Table 4:** Statistics associated with the *Group* × *RAC* × *Device* ANOVA for step length outcome measures.

	Δ_M_				Δ_CV_			
Effect	*SS*	*F*	*p*	η^2^	*SS*	*F*	*p*	η^2^
Device	3.90 × 10^−4^	3.39	.079	< .001	13.74 × 10^0^	25.27	**< .001**	.010
Group	5.90 × 10^−1^	17.67	**< .001**	.416	3.05 × 10^2^	8.49	**.008**	.225
Group × Device	1.82 × 10^−4^	1.58	.221	< .001	5.93 × 10^−1^	1.09	.308	< .001
RAC	4.90 × 10^−2^	28.01	**< .001**	.035	2.09 × 10^1^	2.37	.105	.015
RAC × Device	1.04 × 10^−5^	.26	.771	< .001	2.81 × 10^−1^	.43	.653	< .001
Group × RAC	2.01 × 10^−3^	1.15	.326	.001	3.40 × 10^0^	.39	.681	.003
Group × RAC × Device	8.37 × 10^−5^	2.11	.133	< .001	4.48 × 10^−1^	.69	.508	< .001

“SS” is the portioned sums of squares for each ANOVA term. Significant *p*-values are highlighted in bold.

Both Δ_CV_ measures showed a significant main effect for *Device*, with SmartMOVE showing inflated outcome measure values relative to heel contact–based devices. The corresponding effect sizes, however, were small (η^2^ < .02), as were effect sizes for all other interactions with *Device*.

### 2. *Group* × *Device* ANOVA during self-paced walking

This ANOVA quantified differences between PD and HE during self-paced walking, and whether that effect was significantly influenced by measurement device. ANOVA statistics are presented in Table 5 and Table 6. η^2^ values for *Group* were larger for Δ_M_ statistics (> .41) than Δ_CV_ statistics (< .22). Statistically significant main effects for *Device* were present in step time Δ_CV_, step length Δ_M_, and step length Δ_CV_. However, the corresponding η^2^ values for these significant effects were all small (all η^2^ < .02), as were η^2^ values for all *Group* × *Device* interactions.

**Table 5:** Statistics associated with the *Group* × *Device* ANOVA for step time outcome measures during self-paced walking.

	Δ_M_				Δ_CV_			
Effect	*SS*	*F*	*p*	η^2^	*SS*	*F*	*p*	η^2^
Device	6.16 × 10^−8^	.40	.533	< .0001	1.52 × 10^0^	6.19	.**020**	.0111
Group	2.68 × 10^−2^	15.71	< .**001**	.4166	2.64 × 10^1^	5.64	.**027**	.1931
Group × Device	5.59 × 10^−7^	3.64	.069	< .0001	3.65 × 10^−1^	1.48	.236	.0027

“SS” is the portioned sums of squares for each ANOVA term. Significant *p*-values are highlighted in bold.

**Table 6:** Statistics associated with the *Group* × *Device* ANOVA for step length outcome measures during self-paced walking.

	Δ_M_				Δ_CV_			
Effect	*SS*	*F*	*p*	η^2^	*SS*	*F*	*p*	η^2^
Device	1.63 × 10^−4^	5.09	.**034**	.0004	2.93 × 10^0^	5.88	.**024**	.0064
Group	2.26 × 10^−1^	21.20	< .**001**	.4895	1.00 × 10^2^	6.45	.**019**	.2198
Group × Device	2.18 × 10^−4^	6.82	.**016**	.0005	8.00 × 10^−3^	.02	.900	< .0001

“SS” is the portioned sums of squares for each ANOVA term. Significant *p*-values are highlighted in bold.

### 3. *RAC* × *Device* ANOVA for PD patients

This ANOVA quantified the impact of RAC outcome measures of gait in PD, and whether RAC effects were significantly influenced by measurement device. ANOVA statistics are presented in Table 7 and Table 8. Significant main effects for *RAC* were present in step time Δ_M_, step time Δ_CV_, and step length Δ_M_. Only step time Δ_M_ showed a large (η^2^ = .3067) effect size. All main effects and interactions with *Device* had negligible effect sizes (all η^2^ < .01).

**Table 7:** Statistics associated with the *RAC* × *Device* ANOVA for step time outcome measures in PD patients.

	Δ_M_				Δ_CV_			
Effect	*SS*	*F*	*p*	η^2^	*SS*	*F*	*p*	η^2^
Device	3.54 × 10^−7^	5.50	.**039**	< .0001	2.02 × 10^−1^	.17	.691	.0010
RAC	3.34 × 10^−2^	40.12	< .**001**	.3067	1.80 × 10^1^	6.10	.**008**	.0922
RAC × Device	3.53 × 10^−8^	.13	.883	< .0001	5.81 × 10^−2^	.11	.898	.0003

“SS” is the portioned sums of squares for each ANOVA term. Significant *p*-values are highlighted in bold.

**Table 8:** Statistics associated with the *RAC* × *Device* ANOVA for step length outcome measures in PD patients.

	Δ_M_				Δ_CV_			
Effect	*SS*	*F*	*p*	η^2^	*SS*	*F*	*p*	η^2^
Device	1.96 × 10^−5^	.34	.571	< .0001	4.31 × 10^0^	8.42	.**014**	.0046
RAC	2.87 × 10^−2^	18.32	< .**001**	.0517	1.77 × 10^1^	1.29	.295	.0190
RAC × Device	2.38 × 10^−5^	.84	.444	< .0001	1.13 × 10^−2^	.02	.982	< .0001

“SS” is the portioned sums of squares for each ANOVA term. Significant *p*-values are highlighted in bold.

### 4. *RAC* × *Device* for HE subjects

For sake of completeness, a *RAC* × *Device* ANOVA was performed for HE subject data; ANOVA statistics are presented in Table 9 and Table 10. Main effects for RAC were significant for both step time Δ_M_ and step length Δ_M_, with a substantially larger effect size for step time Δ_M_ (η^2^ = .3586). Unlike the previous ANOVAs, *Device* had a strikingly large effect size for step time Δ_CV_ (η^2^ = .5099), and a small-to-medium effect size for step length Δ_CV_ (η^2^ = .0848). In both cases, *Device* effect sizes were larger than *RAC* effect sizes: outcome measures in HE subjects were more affected by device-induced measurement error than they were by the target RAC manipulation.

**Table 9:** Statistics associated with the *RAC* × *Device* ANOVA for step time outcome measures in HE subjects.

	Δ_M_				Δ_CV_			
Effect	*SS*	*F*	*p*	η^2^	*SS*	*F*	*p*	η^2^
Device	6.24 × 10^−8^	.33	.577	< .0001	5.71 × 10^0^	14.41	.**003**	.5099
RAC	2.63 × 10^−2^	40.97	< .**001**	.3586	3.55 × 10^0^	1.19	.323	.0746
RAC × Device	1.86 × 10^−6^	4.40	.**025**	< .0001	1.52 × 10^−2^	.15	.861	.0135

“SS” is the portioned sums of squares for each ANOVA term. Significant *p*-values are highlighted in bold.

**Table 10:** Statistics associated with the *RAC* × *Device* ANOVA for step length outcome measures in HE subjects.

	Δ_M_				Δ_CV_			
Effect	*SS*	*F*	*p*	η^2^	*SS*	*F*	*p*	η^2^
Device	5.53 × 10^−4^	3.20	.101	.0020	1.00 × 10^1^	17.42	.**002**	.0848
RAC	2.23 × 10^−2^	11.55	< .**001**	.0815	6.55 × 10^0^	1.70	.205	.0555
RAC × Device	7.02 × 10^−5^	1.38	.273	.0003	7.18 × 10^−1^	1.08	.356	.0061

“SS” is the portioned sums of squares for each ANOVA term. Significant *p*-values are highlighted in bold.

## Discussion

The current paper presents the first systematic validation of the accuracy of smartphone-based gait analysis in both Parkinson’s disease (PD) patients and age-matched healthy elderly (HE) subjects. Concurrent validity was obtained for the novel smartphone app (“SmartMOVE”) by simultaneously recording walking patterns using two heel contact–based measurement devices (footswitches to quantify step durations, and a GAITRite sensor walkway to quantify step displacements). Analysis of variance (ANOVA) and eta-squared (η^2^) effect sizes were used to quantify the magnitude of device-related measurement error associated with quantifying differences between PD and HE during self-paced walking, differences between self-paced and metronome-cued walking in PD, and differences between self-paced and metronome-cued walking in HE.

The present study captured two experimental effects frequently noted in the PD literature (see Fig 2). First, during self-paced walking, relative to HE subjects, PD patients walked with slower [57,88,89] and shorter [14,15,88,89] steps, and with increased step time [13,14,90] and step length [88,91,92] variability. Second, when walking with RAC at 110% of self-paced cadence, PD patients walked with faster [15,16,80,81,89] and longer [15,16,79,80] steps, and with decreased step time [15,16,79,80] and step length [15] variability.

The ability to replicate inter-group differences between PD and HE and inter-condition differences between self-paced and externally cued walking serves as a useful manipulation check, allowing the key unknown source of variance—the magnitude of device-related measurement error—to be examined within the context of “typical” experimental results.

### 1. Device-related measurement error in context: Summary

Three summary statements may be offered with respect to patterns η^2^ values among the three target factors: *Group*, *RAC*, and *Device* (cf. Table 3 to Table 10).

The first pattern is defined as a is medium-to-large effect size (η^2^ ≈ .20 or greater) for a target experimental effect (*Group* or *RAC*), and a small effect size (η^2^ < .02) for *Device*. This pattern was observed several times: (1) in all four outcome measures when assessing differences between PD and HE during self-paced walking (Table 5 and Table 6); (2) in step time Δ_M_ when assessing the influence of RAC on gait in PD patients (Table 7); and (3) in step time Δ_M_ when assessing the influence of RAC on gait in HE subjects (Table 9). In these situations, the target experimental manipulation yields a pronounced change that dwarfs device-related measurement error, and indicates a potentially *good* opportunity for SmartMOVE in the clinic.

The second pattern is defined as a small-to-medium effect size (η^2^ ≈ .08) for *RAC*, and a very small (or perhaps “negligible”) effect size (η^2^ < .005) for *Device*. This pattern was observed twice: for step time Δ_CV_ in PD (Table 7), and for step length Δ_M_ in HE (Table 10). Here, the RAC manipulation (self-paced vs. metronome-cued) yielded a less pronounced (though still statistically significant) group-level change, likely due to increased heterogeneity in the way individual subjects responded (as is suggested by the relatively larger error bars). This more complex finding indicates a *possible* opportunity for SmartMOVE in the clinic, with the knowledge that individual differences may contribute more strongly in these cases.

The third pattern is defined as an effect size for *RAC* that is either too similar to *Device* (step length Δ_CV_ in PD; Table 8) or smaller than *Device* (step time Δ_CV_ and step length Δ_CV_ in HE; Table 9 and Table 10). Such a pattern indicates a *poor* opportunity for SmartMOVE in the clinic; that is, conventional gait analysis systems should be used.

Why does Δ_CV_ exhibit greater discrepancies between SmartMOVE and conventional gait analysis, particularly when quantified in HE subjects? A possible explanation for this may be offered.

Step time events were obtained directly from the timestamps of acceleration waveform peaks. By contrast, step length events are derived from machine learning regression models; as such, individual step lengths are associated with stochastic measurement error. Step length estimation involves double integration over the entire waveform segment between detected heel strike analogues (i.e., waveform peaks). These segments contain some degree of noise, due to limited device resolution (i.e., 100-Hz sampling rate) and unintended device movement during walking. Stochastic measurement error on individual steps can, in turn, translate into inflated estimates of step length variability. This effect is particularly noticeable in the case of HE, as those subjects have lower GV to begin with. Measuring RAC-mediated changes in GV in HE subjects (as opposed to PD patients), however, is likely to be of lower clinical interest. As a result, the increased measurement error associated with SmartMOVE in this experimental condition should carry less weight when considering the *overall* utility of SmartMOVE.

The use of effect sizes as an aid to determine “good opportunities” or “possible opportunities” for SmartMOVE in clinical research is just *one* interpretation of the evidence presented in here. Individual clinicians will have unique requirements with respect to the level of measurement accuracy required to make informed decisions about the care and treatment of individual patients. For a clinician or researcher without access (or with limited access) to a conventional gait analysis system, however, SmartMOVE provides the ability to perform quantitative analysis of gait and gait variability or to assess the potential efficacy of an RAC paradigm where *no* opportunity was previously available.

### 2. SmartMOVE-based gait analysis: Caveats and considerations

Three caveats should be noted with respect to choices made in the present experimental methodology, along with our rationale for those choices.

A first caveat relates to the size and clinical characteristics of the present sample. For safety reasons, the inclusion/exclusion criteria for the present study (see Section 1 of the Methods), precluded patients with severe gait dysfunction; most patients in the present sample would be considered to have “moderately advanced” PD. Whether SmartMOVE would perform as well in the case of *severe* gait dysfunction (e.g., shuffling steps or frequent gait freezing episodes) is thus unknown. Quantifying differences between gait or GV in severe PD versus HE, however, would likely have little diagnostic value. Furthermore, patients with severe gait dysfunction are, most likely, poor candidates for home-based, long-term interventions using cueing strategies such as RAC, and thus outside the “target demographic” which would get the most benefit from a tool like SmartMOVE. Nevertheless, a larger and wider sampling across the PD spectrum—within the limits of patient safety—is a valuable future step for this project, both from the perspective of clinical inference and with respect to improving the accuracy and robustness of the machine learning algorithms. Note that the present study’s attrition rate (i.e., yielding samples of 12 PD and 12 HE instead of the target 15 PD and 15 HE) was due to a combination of factors necessitated by stringent methodological requirements (see Section 1 of the Methods), not because smartphone-based gait analysis is inherently more challenging than conventional gait analysis.

A second caveat relates to the placement of SmartMOVE on the body (i.e., affixed at the navel). As reviewed in **Table 1**, previous investigations of smartphone-based gait analysis have positioned the device in a variety of locations; most commonly, over the third lumbar vertebra, as is common in traditional (i.e., non-smartphone) investigations of accelerometry [58,59]. The decision to affix the smartphone on the ventral surface of the body was made with an eye towards two future applications. First, to enhance usability, so that a user (patient) could interact with the device without assistance (e.g., by slipping it in and out of a chest harness). Second, to take advantage of possible camera or video recording applications, which would require that the smartphone’s camera face forwards.

A third caveat relates to the outcome measures calculated by SmartMOVE. In the current paper, we focused on two mathematically straightforward and outcome measures derived from the first-order difference of inter-step times and inter step lengths: Δ_M_ and Δ_CV_, which are widely reported in the clinical literature (e.g., [8–10]). Of course, there are many other outcome measures of gait, which can be summarized in three groups. “Group 1” measures are derived exclusively from a heel strike event series for calculation, such as Δ_M_ and Δ_CV_, as well as detrended fluctuation analysis (e.g., [6,93]). “Group 2” measures require both heel-strike and toe-off events for calculation, and include statistics such as stance or swing time means, SDs, or CVs, or asymmetry values (e.g., [13,94]); and the phase coordination index [95]. “Group 3” measures are derived from frequency-domain analysis of a continuous acceleration signal (as in [61,64,66,67]).

Logically, the optimal outcome measures of GV to investigate are those which best differentiate gait in PD versus gait in HE, or differences in gait between self-paced and metronome-cued walking. A meta-analysis across prior investigations would provide such an answer; unfortunately, no meta-analysis of GV in PD (or PD versus HE) exists. Thus, our choice of step time and step length Δ_M_ and Δ_CV_ (which are all Group 1 measures) was jointly motivated by their prevalence in the clinical literature, as well as by three restrictions imposed by the present experimental design.

First, the practical limitations being able to collect no more than 10 to 12 consecutive steps on a single walk across the 7-m GAITRite mat) precluded the exploration of detrended fluctuation analysis, as there continues to be debate in the literature concerning (1) the degree to which “stitching together” multiple short segments of data violates the assumptions of long-range correlations [96], and (2) the minimum number of gait events (steps or strides) required to reliability differentiate normal versus pathological gait—a number which may be in the hundreds [97,98]. Second, the lack of a clear acceleration waveform analogue to a toe-off event precluded the calculation of Group 2 outcome measures, including the phase coordination index. Third, Δ_M_ and Δ_CV_ measures have been carefully evaluated in terms of their test–retest reliability (as reviewed in [8]), clinical effectiveness (via meta-analysis, as in [26]), and construct validity (via factor analysis, as in [11]; or correlation analysis, as in [83]). No Group 3 measure has been similarly scrutinized.

A final comment related to outcome measures. In the present analyses, care was taken to ensure the same number of gait events across outcome measures (i.e., step time and step length series had the same number of elements per trial) and across devices (i.e., same number of events collected by SmartMOVE and heel contact–based measurement device). We did not, however, control the number of events across *subjects*, other than to set a maximum event count (= 50) per RAC condition. Although there was no systematic difference in number of gait events between groups or across RAC conditions (see S1 Text), increasing the number of events per condition (and setting it as a constant across all subjects) may yield more stable outcome measures, thereby reducing both inter-subject differences and inter-device measurement error.

### 3. SmartMOVE-based gait analysis: Future aims

The present set of results suggests that SmartMOVE offers moderate-to-high accuracy in characterizing differences in first- second-outcome measures of gait between PD patients and HE subjects, and also in characterizing changes in first-moment outcome measures of gait during an RAC paradigm. Three important future research aims emerge directly from these findings.

A first future aim is to improve the stability of device placement on the torso, with an eye towards minimizing errors in step timing and step length estimation. Currently, SmartMOVE is optimized for clinic-based use; i.e., it requires the active participation of a well-trained user to ensure that the quality of collected data is high. Extending the utility and validity of SmartMOVE *outside* the clinic is an important goal.

A second future aim is to explore other situations in which SmartMOVE could be incorporated into the clinic (e.g., characterizing differences between On versus Off medication states, or freezers versus non-freezers), as well as a more a formal assessment of the *classification* accuracy of SmartMOVE relative to heel contact based gait analysis using machine learning techniques [99–101]. A smartphone-based tool with the ability to identify individuals with atypical gait or GV characteristics (e.g., relative to a large sample of age-matched walkers who have previously been tested using smartphone-based gait analysis) would be a highly useful tool in a clinician’s or a physician’s toolbox. In conjunction with this, further work to improve the user interface experience with SmartMOVE (e.g., numeric or graphical representations of data) will be performed.

A third future aim is to evaluate the efficacy of SmartMOVE-enhanced home-based gait training using RAC. RAC has been the focus of numerous previous investigations, systematic reviews [22–24], and meta-analyses [25,26]. For this reason, a detailed exploration of RAC efficacy was not a key feature of the present design, but rather, an illustration of the feasibility of implementing an RAC paradigm on a smartphone. Several previous methods for portable RAC delivery combined with gait analysis have been proposed [50,56,102–104]; all, however, have been designed around the use of footswitches or shoe-mounted IMUs to collect the gait data itself. SmartMOVE captured significant changes in step time Δ_M_ and step time Δ_CV_ in PD with very low device-related measurement error (see Table 5), indicating that these outcome measures in particular could be used to monitor patients’ progress during home-based RAC.

An additional advantage of smartphone-based implementation, in line with the above aim, is the ability to link with commercial streaming music services such as Deezer [105], Rdio [106], or Spotify [107]. Such links would enable the delivery of an RAC paradigm that is *personalized* to an individual patient’s needs (i.e., optimal tempo) and preferences (i.e., favorite musical style or genre). Along these lines, related work from our lab [108,109] has developed a search engine specifically geared towards identifying and retrieving commercial music recordings that maintain a steady tempo—a prerequisite for rhythmic exercise and/or rehabilitation applications. Such personalization is integral to what Boonstra et al. [110] define as the “take home” message with respect to the effective use of cueing paradigms for gait training in PD: that it “should not be prescribed as a ‘one size fits all’ treatment”. An explicit validation of the efficacy of SmartMOVE-based gait training using RAC (relative to “conventional” methods of RAC delivery) is a key future aim.

## Conclusion

As the burden of Parkinson’s disease continues to expand, new strategies and tools—particularly those rooted in telemedicine—will be required to meet it. Here, we describe the foundations of a smartphone-based application, SmartMOVE, that provides clinicians and researchers with a new tool for performing gait analysis: a “hybrid” between the ease and convenience of stopwatch-based assessments and the high accuracy and detail of component-based (sensor plus software plus display) gait analysis systems. In addition, SmartMOVE provides a means to close the loop between the quantification of widely used outcome measures and the delivery of personalized rhythmic auditory cueing—paving the way towards establishing “RAC 2.0”. We hope that technologies like SmartMOVE may one day serve as an accessible tool for the detection of gait dysfunction and an effective nonpharmacological adjuvant for its treatment.

## Supporting Information

S1 Dataset Subject-level outcome measure values for the step time ANOVAs. (XLSX)

S2 Dataset Subject-level outcome measure values for the step length ANOVAs. (XLSX)

S1 Table Group-level means and standard deviations associated with step outcome measures. (DOCX)

S2 Table Group-level means and standard deviations associated with step length outcome measures. (DOCX)

S1 Text Gait analysis preprocessing steps. (PDF)

S2 Text Inter-group, inter-task, and inter-device variance. (PDF)

## References

[1] Dorsey ER, Constantinescu R, Thompson JP, Biglan KM, Holloway RG, Kieburtz K, et al Projected number of people with Parkinson disease in the most populous nations, 2005 through 2030. Neurology. 2007;68: 384 17082464 10.1212/01.wnl.0000247740.47667.03

[2] Achey M, Aldred JL, Aljehani N, Bloem BR, Biglan KM, Chan P, et al The past, present, and future of telemedicine for Parkinson’s disease. Mov Disord. 2014;29: 871–883. 10.1002/mds.25903 24838316

[3] Bloem BR, Hausdorff JM, Visser JE, Giladi N. Falls and freezing of gait in Parkinson’s disease: a review of two interconnected, episodic phenomena. Mov Disord. 2004;19: 871–884. 15300651 10.1002/mds.20115

[4] Giladi N, Hausdorff JM, Balash Y. Episodic and continuous gait disturbances in Parkinson’s disease In: Galvez-Jimenez N, editor. Scientific Basis for the Treatment of Parkinson’s Disease. 2nd ed. Taylor & Francis; 2005 pp. 321–332.

[5] Hausdorff J. Gait variability: methods, modeling and meaning. Journal of NeuroEngineering and Rehabilitation. 2005;2: 19 10.1186/1743-0003-2-19 16033650 PMC1185560

[6] Hausdorff JM. Gait dynamics in Parkinson’s disease: common and distinct behavior among stride length, gait variability, and fractal-like scaling. Chaos. 2009;19: 026113 10.1063/1.3147408 19566273 PMC2719464

[7] Hausdorff JM. Gait dynamics, fractals and falls: finding meaning in the stride-to-stride fluctuations of human walking. Human movement science. 2007;26: 555–589. 17618701 10.1016/j.humov.2007.05.003 PMC2267927

[8] Lord S, Howe T, Greenland J, Simpson L, Rochester L. Gait variability in older adults: a structured review of testing protocol and clinimetric properties. Gait & posture. 2011;34: 443–450. 10.1016/j.gaitpost.2011.07.010 21920755

[9] Hamacher D, Singh NB, Van Dieën JH, Heller MO, Taylor WR. Kinematic measures for assessing gait stability in elderly individuals: a systematic review. Journal of The Royal Society Interface. 2011; 10.1098/rsif.2011.0416 21880615 PMC3203491

[10] Hollman JH, McDade EM, Petersen RC. Normative spatiotemporal gait parameters in older adults. Gait & posture. 2011;34: 111–118. 10.1016/j.gaitpost.2011.03.024 21531139 PMC3104090

[11] Lord S, Galna B, Verghese J, Coleman S, Burn D, Rochester L. Independent Domains of Gait in Older Adults and Associated Motor and Nonmotor Attributes: Validation of a Factor Analysis Approach. The Journals of Gerontology Series A: Biological Sciences and Medical Sciences. 2013;68: 820–827. 10.1093/gerona/gls255 23250001

[12] Verghese J, Holtzer R, Lipton RB, Wang C. Quantitative gait markers and incident fall risk in older adults. The Journals of Gerontology Series A: Biological Sciences and Medical Sciences. 2009;64: 896–901. 10.1093/gerona/glp033 PMC2709543 19349593

[13] Hausdorff JM, Cudkowicz ME, Firtion R, Wei JY, Goldberger AL. Gait variability and basal ganglia disorders: Stride-to-stride variations of gait cycle timing in parkinson’s disease and Huntington’s disease. Movement Disorders. 1998;13: 428–437. 10.1002/mds.870130310 9613733

[14] Baker K, Rochester L, Nieuwboer A. The effect of cues on gait variability: Reducing the attentional cost of walking in people with Parkinson’s disease. Parkinsonism and Related Disorders. 2008;14: 314–320. 17988925 10.1016/j.parkreldis.2007.09.008

[15] Arias P, Cudeiro J. Effects of rhythmic sensory stimulation (auditory, visual) on gait in Parkinson’s disease patients. Experimental Brain Research Experimentelle Hirnforschung Expérimentation Cérébrale. 2008;186: 589–601. 10.1007/s00221-007-1263-y 18214453

[16] Hausdorff JM, Lowenthal J, Herman T, Gruendlinger L, Peretz C, Giladi N. Rhythmic auditory stimulation modulates gait variability in Parkinson’s disease. European Journal of Neuroscience. 2007;26: 2369–2375. 10.1111/j.1460-9568.2007.05810.x 17953624

[17] Lord S, Baker K, Nieuwboer A, Burn D, Rochester L. Gait variability in Parkinson’s disease: an indicator of non-dopaminergic contributors to gait dysfunction? Journal of Neurology. 2010; 10.1007/s00415-010-5789-8 21052710

[18] Bryant MS, Rintala DH, Hou JG, Charness AL, Fernandez AL, Collins RL, et al Gait variability in Parkinson’s disease: influence of walking speed and dopaminergic treatment. Neurological research. 2011;33: 959–964. 10.1179/1743132811Y.0000000044 22080998 PMC5361771

[19] Hausdorff JM, Balash J, Giladi N. Effects of cognitive challenge on gait variability in patients with Parkinson’s disease. Journal of Geriatric Psychiatry and Neurology. 2003;16: 53–58. 12641374 10.1177/0891988702250580

[20] Yogev G, Giladi N, Peretz C, Springer S, Simon ES, Hausdorff JM. Dual tasking, gait rhythmicity, and Parkinson’s disease: Which aspects of gait are attention demanding? European Journal of Neuroscience. 2005;22: 1248–1256. 10.1111/j.1460-9568.2005.04298.x 16176368

[21] Rochester L, Burn DJ, Woods G, Godwin J, Nieuwboer A. Does auditory rhythmical cueing improve gait in people with Parkinson’s disease and cognitive impairment? A feasibility study. Movement Disorders: Official Journal Of The Movement Disorder Society. 2009;24: 839–845. 19199354 10.1002/mds.22400

[22] Rubinstein TC, Giladi N, Hausdorff JM. The power of cueing to circumvent dopamine deficits: a review of physical therapy treatment of gait disturbances in Parkinson’s disease. Movement Disorders. 2002;17: 1148–1160. 12465051 10.1002/mds.10259

[23] Lim I, Van Wegen E, De Goede C, Deutekom M, Nieuwboer A, Willems A, et al Effects of external rhythmical cueing on gait in patients with Parkinson’s disease: a systematic review. Clinical rehabilitation. 2005;19: 695–713. 16250189 10.1191/0269215505cr906oa

[24] Thaut MH, Abiru M. Rhythmic Auditory Stimulation in Rehabilitation of Movement Disorders: A Review Of Current Research. Music Perception. 2010;27: 263–269. 10.1525/mp.2010.27.4.263

[25] de Dreu MJ, van der Wilk ASD, Poppe E, Kwakkel G, van Wegen EEH. Rehabilitation, exercise therapy and music in patients with Parkinson’s disease: a meta-analysis of the effects of music-based movement therapy on walking ability, balance and quality of life. Parkinsonism & Related Disorders. 2012;18 Suppl 1: S114–119. 10.1016/S1353-8020(11)70036-0 22166406

[26] Spaulding SJ, Barber B, Colby M, Cormack B, Mick T, Jenkins ME. Cueing and gait improvement among people with Parkinson’s disease: a meta-analysis. Arch Phys Med Rehabil. 2013;94: 562–570. 10.1016/j.apmr.2012.10.026 23127307

[27] Zatorre RJ, Chen JL, Penhune VB. When the brain plays music: auditory-motor interactions in music perception and production. Nat Rev Neurosci. 2007;8: 547–558. 10.1038/nrn2152 17585307

[28] Schlaug G. The brain of musicians. A model for functional and structural adaptation. Ann N Y Acad Sci. 2001;930: 281–299. 11458836

[29] Thaut MH. Neural basis of rhythmic timing networks in the human brain. Ann N Y Acad Sci. 2003;999: 364–373. 14681157 10.1196/annals.1284.044

[30] Rochester L, Baker K, Nieuwboer A, Burn D. Targeting dopa-sensitive and dopa-resistant gait dysfunction in Parkinson’s disease: selective responses to internal and external cues. Movement disorders : official journal of the Movement Disorder Society. 2011;26: 430–5. 21462258 10.1002/mds.23450

[31] Schaafsma JD, Giladi N, Balash Y, Bartels AL, Gurevich T, Hausdorff JM. Gait dynamics in Parkinson’s disease: relationship to Parkinsonian features, falls and response to levodopa. Journal of the Neurological Sciences. 2003;212: 47–53. 12809998 10.1016/s0022-510x(03)00104-7

[32] Hausdorff J, Nelson M, Kaliton D, Layne J, Bernstein M, Nuernberger A, et al Etiology and modification of gait instability in older adults: a randomized controlled trial of exercise. Journal of applied physiology (Bethesda, Md : 1985). 2001;90: 2117–29. 10.1152/jappl.2001.90.6.2117 11356774

[33] Callisaya ML, Blizzard L, Schmidt MD, Martin KL, McGinley JL, Sanders LM, et al Gait, gait variability and the risk of multiple incident falls in older people: a population-based study. Age Ageing. 2011;40: 481–487. 10.1093/ageing/afr055 21628390

[34] Sterling DA, O’Connor JA, Bonadies J. Geriatric falls: injury severity is high and disproportionate to mechanism. Journal of Trauma and Acute Care Surgery. 2001;50: 116–119. 10.1097/00005373-200101000-00021 11231681

[35] Lord SR, Sherrington C, Menz HB, Close JC. Falls in older people: risk factors and strategies for prevention [Internet]. Cambridge University Press; 2007 Available: https://books.google.com.sg/books?hl=en&lr=&id=1enrvVe81YgC&oi=fnd&pg=PA3&dq=+falls+strategies&ots=7TsTaX9sBr&sig=qOGVIvYXtqzM_b1p8katdaRtCSk

[36] Rubenstein LZ. Falls in older people: epidemiology, risk factors and strategies for prevention. Age and ageing. 2006;35: ii37–ii41. 16926202 10.1093/ageing/afl084

[37] Howcroft J, Kofman J, Lemaire ED. Review of fall risk assessment in geriatric populations using inertial sensors. J Neuroeng Rehabil. 2013;10: 10–91. 23927446 10.1186/1743-0003-10-91 PMC3751184

[38] Ozinga SJ, Machado AG, Miller Koop M, Rosenfeldt AB, Alberts JL. Objective assessment of postural stability in Parkinson’s disease using mobile technology. Movement Disorders. 2015; Available: http://onlinelibrary.wiley.com/doi/10.1002/mds.26214/pdf 10.1002/mds.26214 PMC13059074 25809137

[39] Hausdorff JM, Schaafsma JD, Balash Y, Bartels AL, Gurevich T, Giladi N. Impaired regulation of stride variability in Parkinson’s disease subjects with freezing of gait. Experimental Brain Research Experimentelle Hirnforschung Expérimentation Cérébrale. 2003;149: 187–194. 10.1007/s00221-002-1354-8 12610686

[40] Hove MJ, Suzuki K, Uchitomi H, Orimo S, Miyake Y. Interactive Rhythmic Auditory Stimulation Reinstates Natural 1/f Timing in Gait of Parkinson’s Patients. PloS One. 2012;7: e32600 10.1371/journal.pone.0032600 22396783 PMC3292577

[41] Callisaya ML, Blizzard L, Schmidt MD, McGinley JL, Srikanth VK. Ageing and gait variability—a population-based study of older people. Age and ageing. 2010; afp250. 10.1093/ageing/afp250 20083617

[42] Brach JS, Berlin JE, VanSwearingen JM, Newman AB, Studenski SA. Too much or too little step width variability is associated with a fall history in older persons who walk at or near normal gait speed. J Neuroengineering Rehabil. 2005;2: 21 10.1186/1743-0003-2-21 PMC1187917 16042812

[43] Lemoyne R, Coroian C, Mastroianni T, Grundfest W. Accelerometers for quantification of gait and movement disorders: a perspective review. Journal of Mechanics in Medicine and Biology. 2008;08: 137 10.1142/S0219519408002656

[44] Patel S, Park H, Bonato P, Chan L, Rodgers M. A review of wearable sensors and systems with application in rehabilitation. Journal of neuroengineering and rehabilitation. 2012;9: 21 10.1186/1743-0003-9-21 22520559 PMC3354997

[45] Horak FB, Mancini M. Objective biomarkers of balance and gait for Parkinson’s disease using body-worn sensors. Movement Disorders. 2013;28: 1544–1551. 10.1002/mds.25684 24132842 PMC3927718

[46] Dijkstra B, Kamsma Y, Zijlstra W. Detection of gait and postures using a miniaturised triaxial accelerometer-based system: accuracy in community-dwelling older adults. Age and ageing. 2010;39: 259–262. 10.1093/ageing/afp249 20083616

[47] Weiss A, Herman T, Plotnik M, Brozgol M, Maidan I, Giladi N, et al Can an accelerometer enhance the utility of the Timed Up & Go Test when evaluating patients with Parkinson’s disease? Medical Engineering & Physics. 2010;32: 119–125. 10.1016/j.medengphy.2009.10.015 19942472

[48] Weiss A, Mirelman A, Buchman AS, Bennett DA, Hausdorff JM. Using a Body-Fixed Sensor to Identify Subclinical Gait Difficulties in Older Adults with IADL Disability: Maximizing the Output of the Timed Up and Go. PloS one. 2013;8: e68885 10.1371/journal.pone.0068885 23922665 PMC3726691

[49] Hartmann A, Luzi S, Murer K, de Bie RA. Concurrent validity of a trunk tri-axial accelerometer system for gait analysis in older adults. Gait & Posture. 2009;29: 444–448. 10.1016/j.gaitpost.2008.11.003 19070494

[50] Casamassima F, Ferrari A, Milosevic B, Ginis P, Farella E, Rocchi L. A Wearable System for Gait Training in Subjects with Parkinson’s Disease. Sensors. 2014;14: 6229–6246. 10.3390/s140406229 24686731 PMC4029669

[51] Moore ST, MacDougall HG, Gracies J-M, Cohen HS. Long-term monitoring of gait in Parkinson’s disease. Gait & Posture. 2007;26: 200–207. 10.1016/j.gaitpost.2006.09.011 17046261

[52] Klucken J, Barth J, Kugler P, Schlachetzki J, Henze T, Marxreiter F, et al Unbiased and Mobile Gait Analysis Detects Motor Impairment in Parkinson’s Disease. PLoS ONE. 2013;8: e56956 10.1371/journal.pone.0056956 23431395 PMC3576377

[53] Zhu S, Anderson H, Wang Y. A real-time on-chip algorithm for IMU-Based gait measurement Advances in Multimedia Information Processing–PCM 2012. Springer; 2012 pp. 93–104. Available: http://link.springer.com/chapter/10.1007/978-3-642-34778-8_9

[54] Wagner R, Ganz A. PAGAS: Portable and accurate gait analysis system. Conference proceedings:. Annual International Conference of the IEEE Engineering in Medicine and Biology Society IEEE Engineering in Medicine and Biology Society Conference. 2012;2012: 280–283. doi: 10.1109/EMBC.2012.6345924 23365885

[55] Lord S, Rochester L, Baker K, Nieuwboer A. Concurrent validity of accelerometry to measure gait in Parkinsons Disease. Gait & posture. 2008;27: 357–359. 17604630 10.1016/j.gaitpost.2007.04.001

[56] Bächlin M, Plotnik M, Roggen D, Maidan I, Hausdorff JM, Giladi N, et al Wearable assistant for Parkinson’s disease patients with the freezing of gait symptom. IEEE Transactions on Information Technology in Biomedicine: A Publication of the IEEE Engineering in Medicine and Biology Society. 2010;14: 436–446. 10.1109/TITB.2009.2036165 19906597

[57] Salarian A, Horak FB, Zampieri C, Carlson-Kuhta P, Nutt JG, Aminian K. iTUG, a sensitive and reliable measure of mobility. Neural Systems and Rehabilitation Engineering, IEEE Transactions on. 2010;18: 303–310. 10.1109/TNSRE.2010.2047606 20388604 PMC2922011

[58] Zijlstra W, Hof AL. Assessment of spatio-temporal gait parameters from trunk accelerations during human walking. Gait & posture. 2003;18: 1–10. 10.1016/s0966-6362(02)00190-x 14654202

[59] Zijlstra W. Assessment of spatio-temporal parameters during unconstrained walking. European journal of applied physiology. 2004;92: 39–44. 14985994 10.1007/s00421-004-1041-5

[60] Chan HK., Zheng H, Wang H, Gawley R, Yang M, Sterritt R. Feasibility study on iPhone accelerometer for gait detection. Pervasive Computing Technologies for Healthcare (PervasiveHealth), 2011 5th International Conference on. 2011. pp. 184–187.

[61] How T-V, Chee J, Wan E, Mihailidis A. MyWalk: a mobile app for gait asymmetry rehabilitation in the community. Proceedings of the 7th International Conference on Pervasive Computing Technologies for Healthcare. ICST (Institute for Computer Sciences, Social-Informatics and Telecommunications Engineering); 2013. pp. 73–76. Available: http://dl.acm.org/citation.cfm?id=2534519

[62] LeMoyne R, Mastroianni T, Grundfest W. Wireless accelerometer iPod application for quantifying gait characteristics. Conference Proceedings:. Annual International Conference of the IEEE Engineering in Medicine and Biology Society IEEE Engineering in Medicine and Biology Society Conference. 2011;2011: 7904–7907. 10.1109/IEMBS.2011.6091949 22256173

[63] Mellone S, Tacconi C, Schwickert L, Klenk J, Becker C, Chiari L. Smartphone-based solutions for fall detection and prevention: the FARSEEING approach. Zeitschrift für Gerontologie und Geriatrie. 2012;45: 722–727. 10.1007/s00391-012-0404-5 23184298

[64] Nishiguchi S, Yamada M, Nagai K, Mori S, Kajiwara Y, Sonoda T, et al Reliability and validity of gait analysis by android-based smartphone. Telemedicine Journal and E-Health: The Official Journal of the American Telemedicine Association. 2012;18: 292–296. 10.1089/tmj.2011.0132 22400972

[65] Palmerini L, Mellone S, Rocchi L, Chiari L. Dimensionality reduction for the quantitative evaluation of a smartphone-based Timed Up and Go test. Conference Proceedings:. Annual International Conference of the IEEE Engineering in Medicine and Biology Society IEEE Engineering in Medicine and Biology Society Conference. 2011;2011: 7179–7182. 10.1109/IEMBS.2011.6091814 22255994

[66] Yamada M, Aoyama T, Mori S, Nishiguchi S, Okamoto K, Ito T, et al Objective assessment of abnormal gait in patients with rheumatoid arthritis using a smartphone. Rheumatology International. 2011; 10.1007/s00296-011-2283-2 22193221

[67] Yang M, Zheng H, Wang H, McClean S, Harris N. Assessing the utility of smart mobile phones in gait pattern analysis. Health and Technology. 2012;2: 81–88. 10.1007/s12553-012-0021-8

[68] Zhu S, Ellis RJ, Schlaug G, Ng YS, Wang Y. Validating an iOS-based Rhythmic Auditory Cueing Evaluation (iRACE) for Parkinson’s Disease. Proceedings of the 22nd ACM International Conference on Multimedia. Orlando, FL; 2014. pp. 487–496.

[69] Maetzler W, Domingos J, Srulijes K, Ferreira JJ, Bloem BR. Quantitative wearable sensors for objective assessment of Parkinson’s disease. Movement Disorders. 2013;28: 1628–1637. 10.1002/mds.25628 24030855

[70] Dorsey ER, Venkataraman V, Grana MJ, Bull MT, George BP, Boyd CM, et al Randomized controlled clinical trial of “virtual house calls” for Parkinson disease. JAMA neurology. 2013;70: 565–570. 10.1001/jamaneurol.2013.123 23479138 PMC3791511

[71] Palmerini L, Mellone S, Avanzolini G, Valzania F, Chiari L. Quantification of Motor Impairment in Parkinson’s Disease Using an Instrumented Timed Up and Go Test. IEEE transactions on neural systems and rehabilitation engineering: a publication of the IEEE Engineering in Medicine and Biology Society. 2013; 10.1109/TNSRE.2012.2236577 23292821

[72] Fahn S, Elton R, UPDRS program members. Unified Parkinson’s Disease Rating Scale In: Fahn S, MARSDEN C, Goldstein M, Calne D, editors. Recent Developments in Parkinson’s Disease. Florham Park, NJ: Macmillan Healthcare Information; 1987 pp. 153–163, 293–304.

[73] Goetz CG, Poewe W, Rascol O, Sampaio C, Stebbins GT, Counsell C, et al Movement Disorder Society Task Force report on the Hoehn and Yahr staging scale: status and recommendations. Mov Disord. 2004;19: 1020–1028. 10.1002/mds.20213 15372591

[74] Folstein MF, Folstein SE, McHugh PR. “Mini-mental state”. A practical method for grading the cognitive state of patients for the clinician. J Psychiatr Res. 1975;12: 189–198. 1202204 10.1016/0022-3956(75)90026-6

[75] Giladi N, Shabtai H, Simon ES, Biran S, Tal J, Korczyn AD. Construction of freezing of gait questionnaire for patients with Parkinsonism. Parkinsonism & related disorders. 2000;6: 165–170. 10817956 10.1016/s1353-8020(99)00062-0

[76] Howell DC. The treatment of missing data. The Sage handbook of social science methodology. 2007; 208–224.

[77] Zhu S, Ellis RJ, Schlaug G, Ng YS, Wang Y. Erratum: Validating an iOS-based Rhythmic Auditory Cueing Evaluation (iRACE) for Parkinson’s Disease. [Internet]. 2015 Available: http://www.smcnus.org/wp-content/uploads/2015/05/iRACE-Erratum.pdf

[78] Zhu S, Ellis RJ, Schlaug G, Ng YS, Wang Y. Validating an iOS-based Rhythmic Auditory Cueing Evaluation (iRACE) for Parkinson’s Disease [corrected version] [Internet]. 2015 Available: http://www.smcnus.org/wp-content/uploads/2015/05/iRACE-MM14-corrected.pdf

[79] McIntosh GC, Brown SH, Rice RR, Thaut MH. Rhythmic auditory-motor facilitation of gait patterns in patients with Parkinson’s disease. J Neurol Neurosurg Psychiatry. 1997;62: 22–26. 9010395 10.1136/jnnp.62.1.22 PMC486690

[80] Picelli A, Camin M, Tinazzi M, Vangelista A, Cosentino A, Fiaschi A, et al Three-dimensional motion analysis of the effects of auditory cueing on gait pattern in patients with Parkinson’s disease: a preliminary investigation. Neurological Sciences: Official Journal of the Italian Neurological Society and of the Italian Society of Clinical Neurophysiology. 2010; 10.1007/s10072-010-0228-2 20182896

[81] Freedland R, Festa C, Sealy M, Mcbean A, Elghazaly P, Capan A, et al The effects of pulsed auditory stimulation on various gait measurements in persons with Parkinson’s Disease. NEUROREHABILITATION. 2002;17: 81 12016350

[82] Lord S, Galna B, Coleman S, Burn D, Rochester L. Mild Depressive Symptoms Are Associated with Gait Impairment in Early Parkinson’s Disease. Movement Disorders. 2013; n/a–n/a. 10.1002/mds.25338 23390120

[83] Moe-Nilssen R, Aaslund MK, Hodt-Billington C, Helbostad JL. Gait variability measures may represent different constructs. Gait & Posture. 2010;32: 98–101. 10.1016/j.gaitpost.2010.03.019 20434916

[84] Levine TR, Hullett CR. Eta squared, partial eta squared, and misreporting of effect size in communication research. Human Communication Research. 2002;28: 612–625.

[85] Olejnik S, Algina J. Measures of effect size for comparative studies: Applications, interpretations, and limitations. Contemporary Educational Psychology. 2000;25: 241–286. 10873373 10.1006/ceps.2000.1040

[86] Cohen J. Statistical power analysis for the behavioral sciences [Internet]. 2nd ed. New York: Academic Press; 1988 Available: http://www.sandsresearch.com/White%20Paper%20-%20Optimum%20Participant%20Sample%20Size%20in%20a%20Neuromarketing%20Study%20102009.pdf

[87] Bakeman R. Recommended effect size statistics for repeated measures designs. Behavior research methods. 2005;37: 379–384. 16405133 10.3758/bf03192707

[88] Blin O, Ferrandez AM, Serratrice G. Quantitative analysis of gait in Parkinson patients: increased variability of stride length. Journal of the Neurological Sciences. 1990;98: 91–97. 2230833 10.1016/0022-510x(90)90184-o

[89] Willems AM, Nieuwboer A, Chavret F, Desloovere K, Dom R, Rochester L, et al The use of rhythmic auditory cues to influence gait in patients with Parkinson’s disease, the differential effect for freezers and non-freezers, an explorative study. Disability And Rehabilitation. 2006;28: 721–728. 16809215 10.1080/09638280500386569

[90] Bello O, Marquez G, Camblor M, Fernandez-Del-Olmo M. Mechanisms involved in treadmill walking improvements in Parkinson’s disease. Gait & posture. 2010;32: 118–123. 20452773 10.1016/j.gaitpost.2010.04.015

[91] Lewis GN, Byblow WD, Walt SE. Stride length regulation in Parkinson’s disease: the use of extrinsic, visual cues. Brain. 2000;123 (Pt 10): 2077–2090. 11004125 10.1093/brain/123.10.2077

[92] Bello O, Sánchez JA, Vazquez-Santos C, Fernandez-Del-Olmo M. Spatiotemporal parameters of gait during treadmill and overground walking in Parkinson’s disease. Journal of Parkinson’s disease. 2014;4: 33 10.3233/JPD-130251 24496097

[93] Hausdorff JM, Purdon PL, Peng CK, Ladin Z, Wei JY, Goldberger AL. Fractal dynamics of human gait: stability of long-range correlations in stride interval fluctuations. Journal of Applied Physiology (Bethesda, Md: 1985). 1996;80: 1448–1457. 10.1152/jappl.1996.80.5.1448 8727526

[94] Danoudis M, Iansek R, Simpson P. Freezing of gait in Parkinson’s disease: Further insights into pathophysiological mechanisms. Parkinsonism & Related Disorders. 2012; 10.1016/j.parkreldis.2012.02.005 22397817

[95] Plotnik M, Giladi N, Hausdorff JM. A new measure for quantifying the bilateral coordination of human gait: effects of aging and Parkinson’s disease. Exp Brain Res. 2007;181: 561–570. 10.1007/s00221-007-0955-7 17503027

[96] Kirchner M, Schubert P, Liebherr M, Haas CT. Detrended Fluctuation Analysis and Adaptive Fractal Analysis of Stride Time Data in Parkinson’s Disease: Stitching Together Short Gait Trials. PLoS ONE. 2014;9: e85787 10.1371/journal.pone.0085787 24465708 PMC3900445

[97] Owings TM, Grabiner MD. Measuring step kinematic variability on an instrumented treadmill: how many steps are enough? Journal of biomechanics. 2003;36: 1215–1218. 12831749 10.1016/s0021-9290(03)00108-8

[98] Damouras S, Chang MD, Sejdic E, Chau T. An empirical examination of detrended fluctuation analysis for gait data. Gait & posture. 2010;31: 336–340. 20060298 10.1016/j.gaitpost.2009.12.002

[99] Pogorelc B, Bosnić Z, Gams M. Automatic recognition of gait-related health problems in the elderly using machine learning. Multimedia Tools and Applications. 2012;58: 333–354.

[100] Tahir NM, Manap HH. Parkinson Disease Gait Classification based on Machine Learning Approach. Journal of Applied Sciences. 2012;12: 180–185.

[101] Begg RK, Palaniswami M, Owen B. Support vector machines for automated gait classification. Biomedical Engineering, IEEE Transactions on. 2005;52: 828–838. 10.1109/TBME.2005.845241 15887532

[102] Hove MJ, Iversen JR, Zhang A, Repp BH. Synchronization with competing visual and auditory rhythms: bouncing ball meets metronome. Psychological Research. 2012; 10.1007/s00426-012-0441-0 22638726

[103] Espay AJ, Baram Y, Dwivedi AK, Shukla R, Gartner M, Gaines L, et al At-home training with closed-loop augmented-reality cueing device for improving gait in patients with Parkinson disease. J Rehabil Res Dev. 2010;47: 573–581. 20848370 10.1682/jrrd.2009.10.0165

[104] Mazilu S, Blanke U, Dorfman M, Gazit E, Mirelman A, M. Hausdorff J, et al A Wearable Assistant for Gait Training for Parkinson’s Disease with Freezing of Gait in Out-of-the-Lab Environments. ACM Trans Interact Intell Syst. 2015;5: 5:1–5:31. 10.1145/2701431

[105] Deezer API [Internet]. [cited 1 Nov 2014]. Available: http://developers.deezer.com/

[106] Rdio API [Internet]. 2014 [cited 1 Nov 2014]. Available: http://www.rdio.com/developers/

[107] Spotify API [Internet]. [cited 1 Nov 2014]. Available: https://developer.spotify.com/

[108] Cai Z, Ellis R, Duan Z, Lu H, Wang Y. Basic Exploration of Auditory Temporal Stability (BEATS): A novel rationale, method, and visualization. Proceedings of the 14th International Conference on Music Information Retrieval. 2013. pp. 541–546.

[109] Ellis RJ, Duan Z, Wang Y. Quantifying Auditory Temporal Stability in a Large Database of Recorded Music. PLoS ONE. 2014;9: e110452 10.1371/journal.pone.0110452 25469636 PMC4254286

[110] Boonstra TA, van der Kooij H, Munneke M, Bloem BR. Gait disorders and balance disturbances in Parkinsonʼs disease: clinical update and pathophysiology. Current Opinion in Neurology. 2008;24: 461–471. 10.1097/WCO.0b013e328305bdaf 18607208

