# Supplementary material for: A Validated Smartphone-Based Assessment of Gait and Gait Variability in Parkinson’s Disease
Source: PLoS One. 2015 Oct 30;10(10):e0141694. doi: 10.1371/journal.pone.0141694 (PMC4627774; doi:10.1371/journal.pone.0141694)
Supplement: S1 Table — (DOCX) [file pone.0141694.s003.docx]

**S1 Table.** Group-level means and standard deviations associated with *step time*
outcome measure ANOVAs (Fig. 2a).

|  |  |  |  | **Δ_M_** | |  | **Δ_CV_** | |  |  | |  |  | |
| --- | --- | --- | --- | --- | --- | --- | --- | --- | --- | --- | --- | --- | --- | --- |
| **Device** | **Group** | **Condition** |  | **M** | **SD** |  | **M** | **SD** |  |  |  |  |  |  |
|  |  |  |  |  |  |  |  |  |  |  |  |  |  |  |
| SmartMOVE | PD | Self-paced |  | 0.542 | 0.029 |  | 5.584 | 1.541 |  |  |  |  |  |  |
|  |  | 100% RAC |  | 0.529 | 0.033 |  | 4.540 | 1.336 |  |  |  |  |  |  |
|  |  | 110% RAC |  | 0.491 | 0.029 |  | 4.401 | 1.365 |  |  |  |  |  |  |
|  |  |  |  |  |  |  |  |  |  |  |  |  |  |  |
|  | HC | Self-paced |  | 0.495 | 0.029 |  | 4.275 | 1.541 |  |  |  |  |  |  |
|  |  | 100% RAC |  | 0.493 | 0.033 |  | 4.056 | 1.336 |  |  |  |  |  |  |
|  |  | 110% RAC |  | 0.453 | 0.029 |  | 3.745 | 1.365 |  |  |  |  |  |  |
|  |  |  |  |  |  |  |  |  |  |  |  |  |  |  |
| GaitRITE | PD | Self-paced |  | 0.542 | 0.029 |  | 5.403 | 1.600 |  |  |  |  |  |  |
|  |  | 100% RAC |  | 0.530 | 0.033 |  | 4.450 | 1.168 |  |  |  |  |  |  |
|  |  | 110% RAC |  | 0.491 | 0.029 |  | 4.355 | 1.130 |  |  |  |  |  |  |
|  |  |  |  |  |  |  |  |  |  |  |  |  |  |  |
|  | HC | Self-paced |  | 0.495 | 0.029 |  | 3.744 | 1.600 |  |  |  |  |  |  |
|  |  | 100% RAC |  | 0.493 | 0.033 |  | 3.455 | 1.168 |  |  |  |  |  |  |
|  |  | 110% RAC |  | 0.453 | 0.029 |  | 3.187 | 1.130 |  |  |  |  |  |  |
