# Supplementary material for: A Validated Smartphone-Based Assessment of Gait and Gait Variability in Parkinson’s Disease
Source: PLoS One. 2015 Oct 30;10(10):e0141694. doi: 10.1371/journal.pone.0141694 (PMC4627774; doi:10.1371/journal.pone.0141694)
Supplement: S2 Table — (DOCX) [file pone.0141694.s004.docx]

**S2 Table.** Group means and standard deviations associated with *step length*
outcome measure ANOVAs (Fig. 2b).

|  |  |  |  | **Δ_M_** | |  | **Δ_CV_** | |  |  | |  |  | |
| --- | --- | --- | --- | --- | --- | --- | --- | --- | --- | --- | --- | --- | --- | --- |
| **Device** | **Group** | **Condition** |  | **M** | **SD** |  | **M** | **SD** |  |  |  |  |  |  |
|  |  |  |  |  |  |  |  |  |  |  |  |  |  |  |
| SmartMOVE | PD | Self-paced |  | 0.508 | 0.072 |  | 7.788 | 2.691 |  |  |  |  |  |  |
|  |  | 100% RAC |  | 0.529 | 0.077 |  | 7.732 | 3.222 |  |  |  |  |  |  |
|  |  | 110% RAC |  | 0.555 | 0.077 |  | 6.685 | 2.175 |  |  |  |  |  |  |
|  |  |  |  |  |  |  |  |  |  |  |  |  |  |  |
|  | HC | Self-paced |  | 0.641 | 0.072 |  | 4.876 | 2.691 |  |  |  |  |  |  |
|  |  | 100% RAC |  | 0.646 | 0.077 |  | 4.610 | 3.222 |  |  |  |  |  |  |
|  |  | 110% RAC |  | 0.683 | 0.077 |  | 4.374 | 2.175 |  |  |  |  |  |  |
|  |  |  |  |  |  |  |  |  |  |  |  |  |  |  |
| Biometrics | PD | Self-paced |  | 0.507 | 0.075 |  | 7.269 | 2.960 |  |  |  |  |  |  |
|  |  | 100% RAC |  | 0.531 | 0.080 |  | 7.242 | 3.008 |  |  |  |  |  |  |
|  |  | 110% RAC |  | 0.557 | 0.079 |  | 6.227 | 2.401 |  |  |  |  |  |  |
|  |  |  |  |  |  |  |  |  |  |  |  |  |  |  |
|  | HC | Self-paced |  | 0.649 | 0.075 |  | 4.408 | 2.960 |  |  |  |  |  |  |
|  |  | 100% RAC |  | 0.652 | 0.080 |  | 3.768 | 3.008 |  |  |  |  |  |  |
|  |  | 110% RAC |  | 0.686 | 0.079 |  | 3.446 | 2.401 |  |  |  |  |  |  |
