# Supplementary material for: A Validated Smartphone-Based Assessment of Gait and Gait Variability in Parkinson’s Disease
Source: PLoS One. 2015 Oct 30;10(10):e0141694. doi: 10.1371/journal.pone.0141694 (PMC4627774; doi:10.1371/journal.pone.0141694)
Supplement: S1 Text — (PDF) [file pone.0141694.s005.pdf]

# S1 Text: Gait analysis preprocessing steps

SmartMOVE preprocessing can be summarized in nine steps, with cross-references to our previous technical paper [1].

## Step 1: IMU preprocessing

IMU preprocessing comprises (a) waveform interpolation and resampling to 1000 Hz, (b) straight-versus-turning walking path segmentation (only straight path walking was analyzed presently), (c) device tilt compensation, and (d) low-pass filtering (cf. [1] Section 5.1).

## Step 2: Synchronization of device clocks

Inter-device synchronization to align the clocks SmartMOVE, Biometrics, and GAITRite together. Precise alignment was critical to evaluate the temporal error between IMU waveform events (i.e, peaks and troughs) and true heel strike times (measured by the heel-mounted footswitch sensors) (cf. [1] Section 5.2).

## Step 3: Waveform annotation

Manual annotation of waveform peaks and troughs in the AP and UD channel, which would serve as input to subsequent machine learning operations. Previous accelerometry-based gait analysis methods have used waveform peaks (i.e., local maxima) in the AP channel and/or UD channel as analogues of physical heel strike (HS) events. However, these methods were primarily focused on gait analysis in *healthy* individuals, and have not been validated for gait analysis in PD patients.

**Fig. S1** presents a 3.5-second excerpt of AP waveform data from a single subject, highlighting the location of manually annotated waveform peaks ($t_P$; indicated by down arrows) and troughs ($t_T$; indicated by up arrows) with respect to the temporal location of actual strike events ($t_{HS}$; vertical lines). Notably, $t_P$ and $t_T$ values exhibit both an overall delay from the corresponding $t_{HS}$, as well as step-to-step variability in the amount of delay. Here, we use $t_\varepsilon$ to indicate temporal error between a given $t_{HS}$ and the associated waveform events: $t_{\varepsilon,P} = t_P - t_{HS}$, and $t_{\varepsilon,T} = t_T - t_{HS}$. Logically, the *spread* of $t_\varepsilon$-values rather than the *average* $t_\varepsilon$-value is the more relevant statistic with respect to outcome measure calculation. A high spread of $t_\varepsilon$-values indicates a greater amount of signed temporal error from step to step, which is likely to yield into inflated estimates of gait variability. Put another way, if a $t_P$ or $t_T$ event series were simply shifted in time (i.e., by adding constant temporal error) relative to another a $t_{HS}$ event series, any statistics based on *inter-event* intervals within each series would remain identical.

## Step 4: Analysis of signed temporal errors

To assess which waveform event was the most temporally stable analogue of actual heel strikes, a set of $t_\varepsilon$-values was calculated for each subject in each RAC condition (aggregated across all walks, and ignoring the turn; cf. Fig. 1d in the main text) and each IMU waveform event (AP peaks, AP troughs, UD peaks, and UD troughs). For each set of $t_\varepsilon$-values, the *interdecile range* (IDR; a robust measure of spread, calculated as the difference between the 90$^{th}$ and 10$^{th}$ percentiles of observed values) was taken. **Fig. S1b** presents data from a single subject; the set of $t_\varepsilon$-values associated with AP peaks (squares) has a narrower IDR than the set of $t_\varepsilon$-values associated with AP troughs (circles).

IDR values were then analyzed in a 2 (*Group*: PD vs. HE) × 2 (*Channel*: AP or UD) × 2 (*Waveform Event*: Peak or Trough) ANOVA, with results visualized in **Fig. S1c** (error bars reflect standard errors). **Table S1** summarizes statistics associated with main effects and interactions.

Based on our previous study (i.e., Figure 3d in [1]), we expected a significant *Channel* × *Event* interaction, reflecting both a main effect for *Channel* (i.e., IDRs for UD events were larger than IDRs for AP events) and a main effect for *Event* (i.e., IDRs for waveform peaks smaller than IDRs for waveform troughs). The present results (**Fig. S1** and **Table S1**) confirm this overall finding. More importantly, however, the nonsignificant three-way interaction of *Channel*, *Event*, and *Group* ($p$ = .090) indicates that the *Channel* × *Event* interaction was not significantly different between the PD patient sample and the HE subject sample. In both groups, AP waveform peaks had the lowest temporal error for both PD and HE. This finding supports the use of AP peaks as the most temporally stable analogue of true heel strikes. AP peaks were thus used to train the machine learning algorithms (Step 5 in Section 3 of the Methods) and calculate the resultant step time and step length outcome measures (Step 10 in Section 3 of the Methods).

An important correction must be noted to our previous report [1], which stated that AP waveform *troughs* were the most temporally stable waveform event. In fact, an error during preprocessing resulting in a flipping of raw waveform data about the *x*-axis, resulting in waveform peaks appearing as waveform troughs; as shown, for example, in Zhu et al., Figure 3b. This error has been corrected in the analysis and text presented above. (An erratum [2] and corrected manuscript [3] are also available.)

*(Figure S1 and Table S1 appear on the next page)*

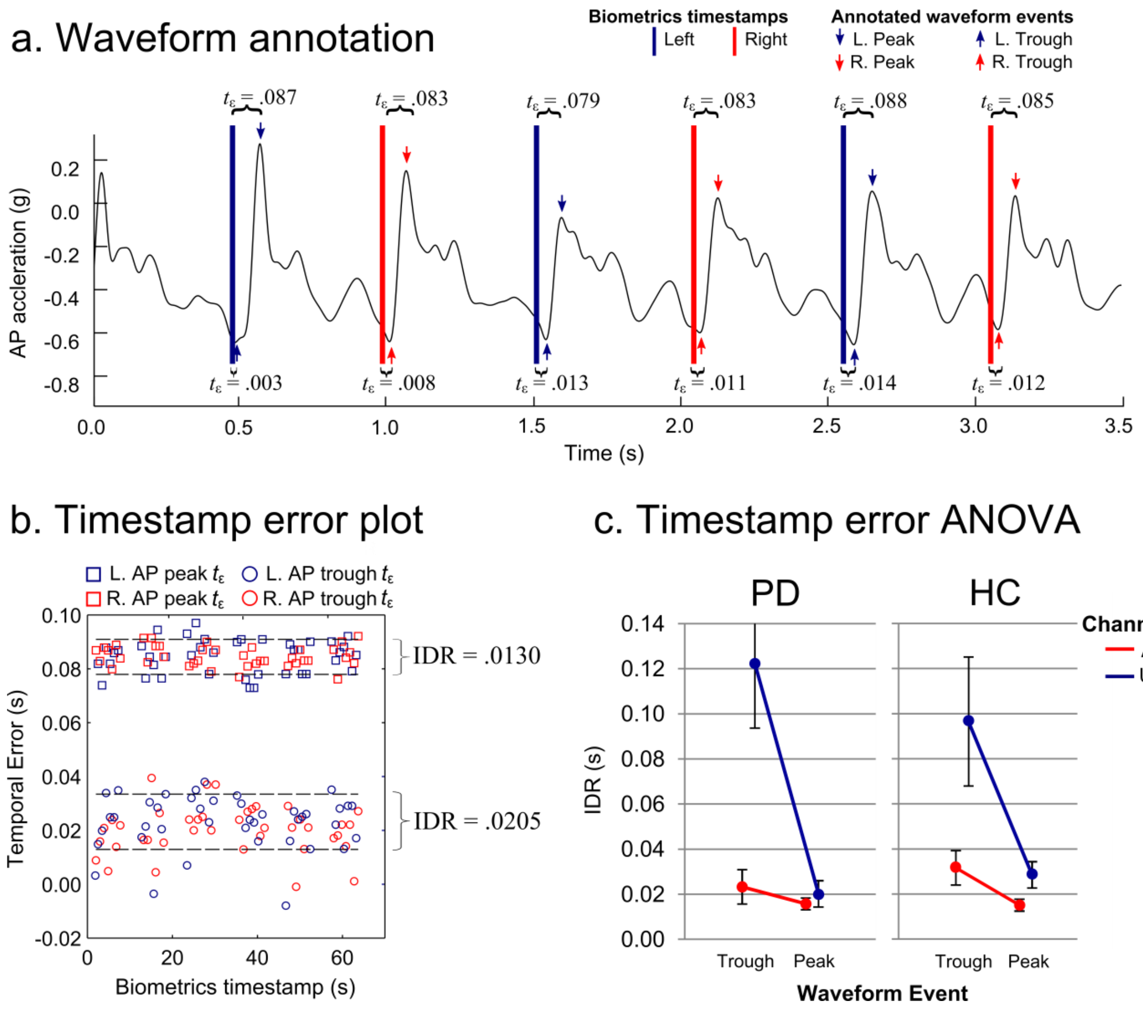


**Fig. S1. Assessing the temporal stability of IMU waveform events.** (a) An excerpt of AP channel accelerometer data, highlighting the temporal error ($t_\varepsilon$) of waveform peaks (marked by down arrows) and troughs (up arrows) relative to actual heel strikes (vertical lines). (b.) The spread of $t_\varepsilon$-values was quantified using the interdecile range (IDR), illustrated here for a single subject. (c.) Means and standard deviations of IDR values, split by group (PD. vs. HE), IMU channel (AP vs. UD), and waveform event (Peak or Trough).

**Table S1. ANOVA statistics.**

| | SS | *F* | *p* | $\eta^2$ |
|---|---|---|---|---|
| Group | $1.42 \times 10^{-4}$ | .16 | .695 | .0006 |
| Channel | $4.96 \times 10^{-2}$ | 54.41 | < .001 | .2216 |
| Channel × Group | $9.42 \times 10^{-4}$ | 1.03 | .320 | .0042 |
| Event | $5.66 \times 10^{-2}$ | 57.74 | < .001 | .2527 |
| Event × Group | $9.48 \times 10^{-4}$ | 0.97 | .335 | .0042 |
| Channel × Event | $3.20 \times 10^{-2}$ | 36.22 | < .001 | .1429 |
| Channel × Event × Group | $2.78 \times 10^{-3}$ | 3.15 | .090 | .0124 |

"SS" is the partitioned sums of squares.

## Step 5:  Machine-learning operations

Machine learning was used to perform (a) heel strike analogue detection (i.e., distinguishing AP peaks associated with heel strike from other local waveform minima; (b) step length calculation; and (c) left-versus-right foot identification.

A total of 7,677 true heel strikes (i.e., collected by the Biometrics footswitch sensors) and 30,479 manually annotated AP peaks (7,677 HS peaks and 22,802 non-HS peaks) were combined across all Walking Evaluations of all 24 subjects. Based on 10-fold cross-validation, our heel strike detection algorithm (cf. [1], Section 5.4) achieved a precision (i.e., positive predictive value) and recall (i.e., sensitivity) both equal to 1.0.  If the binary output from the classifier (i.e., a decision of "heel strike event" or "not a heel strike event", using a decision boundary of 0.5) were obtained without further rule-based refinement, the precision and recall were .9998 and .9874, respectively.

On the same heel strike dataset, our left-vs.-right foot identification algorithm (cf. [1], Section 5.6) achieved 100% accuracy.

Finally, a step length dataset of 3,372 steps was combined across all walks of all subjects with ground truth step lengths collected from GAITRite. The root mean square error (RMSE) of our step length calculation algorithm (cf. [1], Section 5.5) was 3.57 cm based on 10-fold cross-validation, or 6.08% when expressed as a CV value (i.e., RMSE divided by mean step length).

## Step 6: Event series creation

Step 6: Creation of two *event series*: a step time event series comprising successive timestamps (in seconds, relative to the starting time of the trial), and a step length event series comprising successive displacement values (in cm, relative to the starting position of the trial).

## Step 7: Gait event outlier detection

Step 7: Outlier detection on the step time event series to identify inter-event intervals that are suspiciously long (e.g., due to a missed heel strike within a single trial) or suspiciously short (e.g., due to a spurious "double heel strike").

A simple "successive percentage change" (SPC) transformation was used to detect inter-event interval *outliers*: inter-event intervals that are suspiciously long (e.g., due to a missed heel strike within a single trial) or suspiciously short (e.g., due to a spurious "double heel strike").  First, the first-order difference of a step time or step length event series was taken, creating an inter-event

series (IES), as highlighted in Fig. S1a. Second, a series of SPC values is defined using successive pairs of IES values:

$$\mathrm{SPC}_i = 100 \times \frac{|\mathrm{IES}_{i+1} - \mathrm{IES}_i|}{\mathrm{IES}_i}.$$

If two successive IES values ($\mathrm{IES}_i$ and $\mathrm{IES}_{i+1}$) are identical, for example, then $\mathrm{SPC}_i$ will have a value of 0. Next, for any $S_{\mathrm{SPC},i} \geq 50$ (i.e., a change greater than ±50% from interval $i$ to interval $i$+1), the associated event series element (that is, element $i$ + 2) was flagged as an outlier. For example, in Fig. S1a, the very long $\mathrm{IES}_9$ (roughly double the value of $\mathrm{IES}_8$ due to a missed heel strike) will yield an SPC that is ≈ 200, far greater than 50. As a result, a "break point" would be inserted that isolates events $e_1$ through $e_9$ as a contiguous event series.

## Step 8: Δ-series creation

The first-order difference of "outlier free" segments of data will be referred to as a "Δ-series" (Fig. S1b).

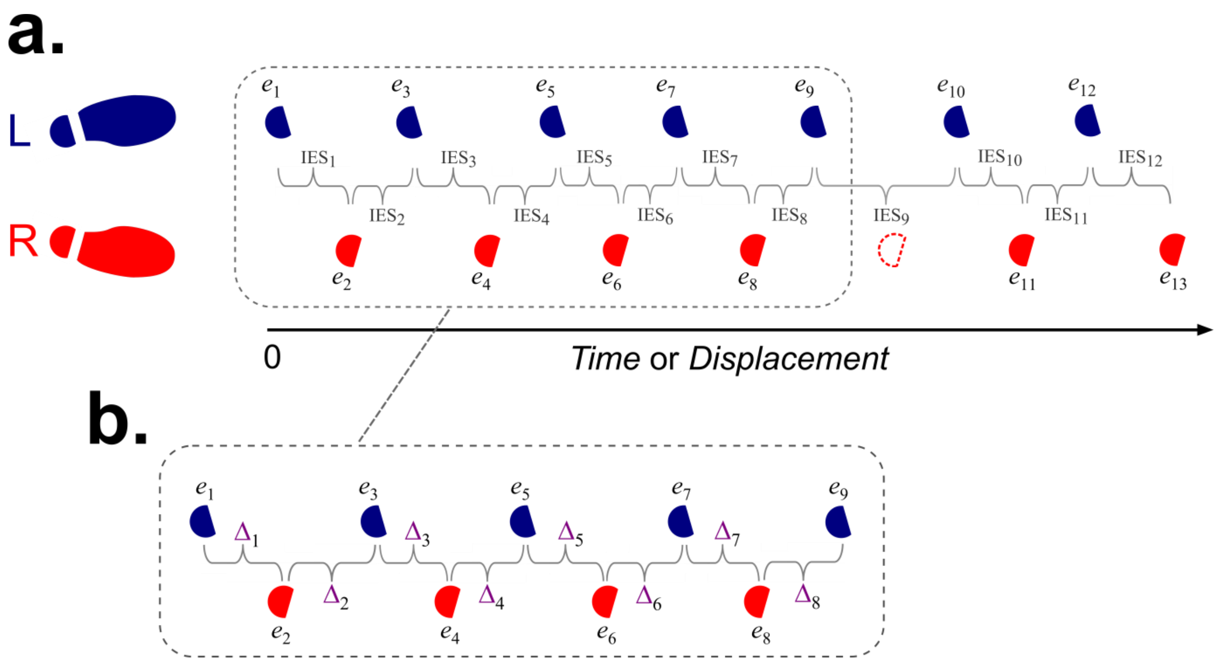


**Fig. S1. Outlier detection.** (a.) A hypothetical heel strike series in which a missing event is present. (b). The outlier-free event series is then subjected to a first-order difference transformation (Δ-series).

## Step 9: Δ-series concatenation

Concatenation of Δ-series across all trials within the same RAC condition (as in Lord et al. [4,5]), so as to provide more stable outcome measures. For example, if the self-paced condition four trials that yielded 10 Δ-values, those Δ-values were concatenated into a single set of 40 Δ-values prior to outcome measure calculation. Concatenated Δ-series with more than 50 elements were truncated at 50. Importantly, the number of elements in the final Δ-series for a given subject and RAC condition will always be *identical* for SmartMOVE and the heel contact–based device; thus, any differences in outcome measures will not be due to a difference in the number of inter-event intervals. Furthermore, for each trial, step time events which occurred before the first step recorded on GAITRite or after the last step recorded by GAITRite were excluded prior to outcome measure calculation. The average number of events per subject per RAC condition was 36 (SD = 10).

To determine whether the number of inter-event intervals concatenated across all trials in a given RAC condition were significantly different among RAC conditions or between PD and HE groups, a *Group* (2 levels: PD and HE) × *RAC* (3 levels: Self-paced, 110%, 110%) ANOVA was performed. Main effects were nonsignificant for *Group* [$F(1,22) = 1.61$, $p = .231$], RAC [$F(2,22) = .63$, $p = .541$] and their interaction [$F(2,22) = .40$, $p = .679$], indicating a lack of

## References


1. Zhu S, Ellis RJ, Schlaug G, Ng YS, Wang Y. Validating an iOS-based Rhythmic Auditory Cueing Evaluation (iRACE) for Parkinson's Disease. Proceedings of the 22nd ACM International Conference on Multimedia. Orlando, FL; 2014. pp. 487–496.

2. Zhu S, Ellis RJ, Schlaug G, Ng YS, Wang Y. Erratum: Validating an iOS-based Rhythmic Auditory Cueing Evaluation (iRACE) for Parkinson's Disease. [Internet]. 2015. Available: http://www.smcnus.org/wp-content/uploads/2015/05/iRACE-Erratum.pdf

3. Zhu S, Ellis RJ, Schlaug G, Ng YS, Wang Y. Validating an iOS-based Rhythmic Auditory Cueing Evaluation (iRACE) for Parkinson's Disease [corrected version] [Internet]. 2015. Available: http://www.smcnus.org/wp-content/uploads/2015/05/iRACE-MM14-corrected.pdf

4. Lord S, Baker K, Nieuwboer A, Burn D, Rochester L. Gait variability in Parkinson's disease: an indicator of non-dopaminergic contributors to gait dysfunction? J Neurol. 2010; doi:10.1007/s00415-010-5789-8

5. Lord S, Galna B, Coleman S, Burn D, Rochester L. Mild Depressive Symptoms Are Associated with Gait Impairment in Early Parkinson's Disease. Mov Disord. 2013; 28; 634–639. doi:10.1002/mds.25338
