# Supplementary material for: A Validated Smartphone-Based Assessment of Gait and Gait Variability in Parkinson’s Disease
Source: PLoS One. 2015 Oct 30;10(10):e0141694. doi: 10.1371/journal.pone.0141694 (PMC4627774; doi:10.1371/journal.pone.0141694)
Supplement: S2 Text — (PDF) [file pone.0141694.s006.pdf]

S2 Text: Inter-group, inter-task, and inter-device variance

Three sources of variance are important to consider in the present experimental design: inter-device variance (e.g., differences in outcome measure values between the "standard" measurement device and the "novel" measurement device); inter-group variance (e.g., differences in outcome measures between PD and HE, or differences between "On" and "Off" medication states); and inter-task variance (e.g., differences in outcome measures associated with task presence or task difficulty). The *context* in which inter-device error emerges is important to capture, and the *method* used to quantify it is an important consideration.

For example, although the Limits of Agreement on a Bland–Altman plot [1,2] can quantify variance due to inter-device differences, they give no insight into *expected* sources of variance: differences between groups (e.g., PD vs. HE), medication states ("On" versus "Off"), treatment conditions (e.g., treatment present versus treatment absent), task difficulty levels (e.g., single- versus dual-task), and so on. Likewise, although intraclass correlation coefficients are used to quantify inter-rater reliability [3] or test-retest reliability [4], they are statistically inappropriate for assessing inter-device agreement. Devices—unlike raters or test occasions—have a fixed rather than a random assignment [5,6].

If on some measurement scale, for example, it were found that PD and HE differed by 10 points on average, or treatment-present versus treatment-absent conditions differed by 5 points on average, then an observed inter-device measurement error of ±1 point may be deemed "acceptable" by a clinician—particularly if the novel measurement device offered *other* advantages over the standard measurement device (e.g., portability, convenience, cost, etc.).

In the present study, device-related measurement error associated with SmartMOVE versus heel contact–based gait measurement was assessed in the context of two well-studied experimental effects: differences in outcome measures between PD and HE and differences in outcome measures between self-paced versus metronome-cued walking conditions. Analysis of variance (ANOVA) was used to assess inter-group, inter-condition, and inter-device differences simultaneously. By partitioning the variance observed in the data into an underlying set of underlying sources, ANOVA thus enables a comparison of the amount of "undesirable" variance due to device-related measurement error (here, between SmartMOVE versus heel contact–based devices) relative to the amount of "desirable" variance due to inter-group effects (here, Parkinson's versus healthy) and inter-condition effects (here, self-paced walking versus walking with RAC). (Inter-group and inter-condition variance are "desirable" in that the greater the difference in scores between groups or between conditions, the greater the discriminative ability of a given test instrument.)

References

[1] Bland JM, Altman D. Statistical methods for assessing agreement between two methods of clinical measurement. The lancet. 1986;327: 307–310.

[2] Bland JM, Altman DG. Measuring agreement in method comparison studies. Statistical Methods in Medical Research. 1999;8: 135. doi:10.1191/096228099673819272

[3] Shrout PE, Fleiss JL. Intraclass correlations: Uses in assessing rater reliability. Psychological Bulletin. 1979;86: 420–428. doi:10.1037/0033-2909.86.2.420

[4] Weir JP. Quantifying test-retest reliability using the intraclass correlation coefficient and the SEM. The Journal of Strength & Conditioning Research. 2005;19: 231–240.

[5] Bland JM, Altman DG. A note on the use of the intraclass correlation coefficient in the evaluation of agreement between two methods of measurement. Computers in biology and medicine. 1990;20: 337–340.

[6] Bland JM, Altman DG. Comparing two methods of clinical measurement: a personal history. International Journal of Epidemiology. 1995;24: S7–S14.
